# Impact of different supply air and recirculating air filtration systems on stable climate, animal health, and performance of fattening pigs in a commercial pig farm

**DOI:** 10.1371/journal.pone.0194641

**Published:** 2018-03-20

**Authors:** Cindy Wenke, Janina Pospiech, Tobias Reutter, Bettina Altmann, Uwe Truyen, Stephanie Speck

**Affiliations:** 1 Institute of Animal Hygiene and Veterinary Public Health, University of Leipzig, Leipzig, Germany; 2 REVENTA^®^ GmbH, Horstmar, Germany; 3 Institute for Medical Informatics, Statistics and Epidemiology, University of Leipzig, Leipzig, Germany; The Ohio State University, UNITED STATES

## Abstract

Biosecurity is defined as the implementation of measures that reduce the risk of disease agents being introduced and/or spread. For pig production, several of these measures are routinely implemented (e.g. cleaning, disinfection, segregation). However, air as a potential vector of pathogens has long been disregarded. Filters for incoming and recirculating air were installed into an already existing ventilation plant at a fattening piggery (3,840 pigs at maximum) in Saxony, Germany. Over a period of three consecutive fattening periods, we evaluated various parameters including air quality indices, environmental and operating parameters, and pig performance. Animal data regarding respiratory diseases, presence of antibodies against influenza A viruses, PRRSV, and *Actinobacillus pleuropneumoniae* and lung health score at slaughter were recorded, additionally. There were no significant differences (p = 0.824) in total bacterial counts between barns with and without air filtration. Recirculating air filtration resulted in the lowest total dust concentration (0.12 mg/m^3^) and lung health was best in animals from the barn equipped with recirculating air filtration modules. However, there was no difference in animal performance. Antibodies against all above mentioned pathogens were detected but mostly animals were already antibody-positive at re-stocking. We demonstrated that supply air filtration as well as recirculating air filtration technique can easily be implemented in an already existing ventilation system and that recirculating air filtration resulted in enhanced lung health compared to supply air-filtered and non-filtered barns. A more prominent effect might have been obtained in a breeding facility because of the longer life span of sows and a higher biosecurity level with air filtration as an add-on measure.

## Introduction

In today’s pig industry with facilities of large animal numbers specific biosecurity implementations are a prerequisite to guarantee animal health and performance. Standard biosecurity protocols mainly include measures to prevent infections via direct or indirect transmission routes like pigs, semen, human beings, transport vehicles, insects, and fomites. However, treatment of air is often not included in these measures. Air filtration as a biosecurity measure can be used in different fields of application. Supply air filtration has already been demonstrated to efficiently reduce porcine reproductive and respiratory syndrome virus (PRRSV) and *Mycoplasma* (*M*.) *hyopneumoniae* in incoming air [1–6] and numerous sow farms in swine dense areas in the Midwest United States implemented this technique [7]. The concept of filtering indoor air in pig production [8–11] is still new to pig production facilities [12]. Indoor airborne particles and microorganisms mainly originate from animals, food, and bedding [13]. Particles with aerodynamic diameters >2.5 μm and <10 μm have an acute effect on respiratory health, particularly among susceptible populations [14]. Pathogens are often attached to dust particles [15,16] and this may lead to a faster spread in and between animal buildings. Furthermore, dust may impair the respiratory tract thus increasing susceptibility to infections with high and low pathogenic microorganisms [17,10]. Recently, methicillin-resistant *Staphylococcus aureus* (MRSA) has been detected bound to dust particles and in high concentrations in the air of pig facilities [18,19] posing a health threat to animals and caretakers [20,21]. Hence, indoor air quality can also be improved by filtering dust and its associated hazards. A high filtering efficacy (92.0%-99.9%) was demonstrated using two different air filter types for PRRSV, *Staphylococcus aureus* and *Actinobacillus pleuropneumoniae* at laboratory scale [22]. Filters consisted either of an EU class F9 (MERV 16) fiberglass filter combined to an upstream prefilter (EU class G4, MERV 6–8), or of a glass wool filter mat (EU class F8-9, MERV 14–16). The above mentioned air filter types were installed into an already existing ventilation plant at a fattening piggery in Saxony, Germany. Over a period of three consecutive fattening periods, various parameters including air quality indices (total airborne dust levels, bacteria counts, ammonia, and CO_2_ levels), environmental and operating parameters (temperature, ventilation air flow, relative humidity), and pig performance were monitored. In addition, animal data regarding respiratory diseases, presence of antibodies against selected microorganisms as well as lung health scoring at slaughter were recorded.

The objective of this study was to evaluate the impact of three different mechanical air filtrations systems on air quality, animal health and animal performance in comparison to a non-filtered control. Two of these systems were designed for supply air filtration and a third for recirculating air filtration.

## Material and methods

### General description of the pig facility

**Fig 1:**
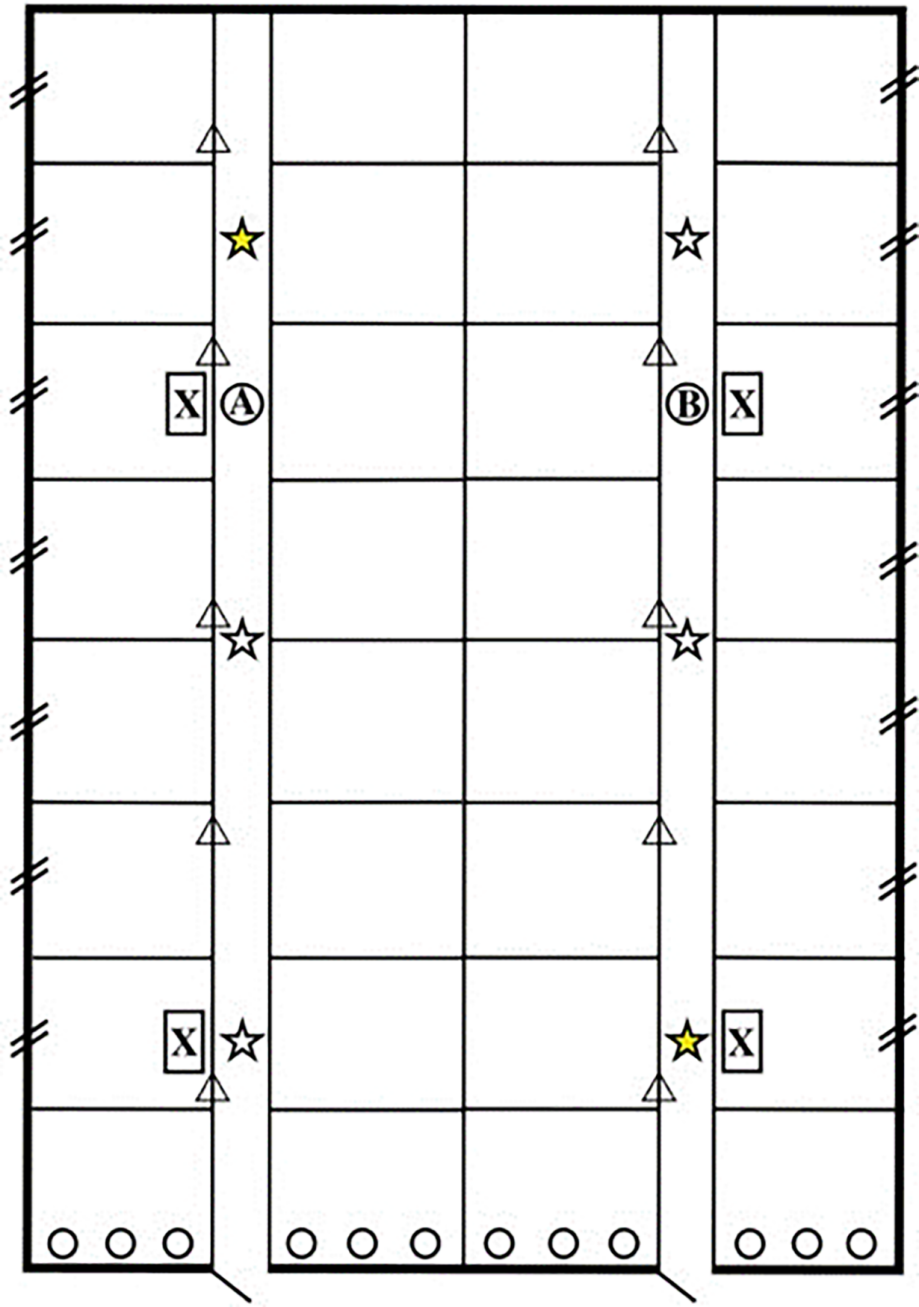
Structure of the four barns and sampling points. Each barn consisted of 32 pens and was accessible via two doors. Exhaust air outlets in each barn are given as circles. Slashes on both sides of the picture symbolize the fresh air inlets of barn 3 and 4. Triangles indicate the fresh air distributors of barn 1. Dust was measured at two sampling locations that were randomly selected on the day of sampling but were identical with the sampling points for air sampling. The latter sampling points (n = 6) are indicated by stars. Stars in yellow indicate sampling points for ammonia (NH_3_). A (barn 1 and 3) and B (barn 2 and 4) represent the positions of the sensors for CO_2_ and relative humidity. Recirculating air filter modules in barn 4 are represented by a framed X.

The study was conducted at a pig farm located in Saxony (Germany) over three fattening periods (thirteen months) during 2015–2016. The test facility on the farm was composed of four identical barns, which housed a maximum of 960 pigs each. Each barn (50 m long, 21.7 m wide) consisted of 32 pens with a maximum of 32 pigs each, housed on fully slatted floors without litter. Slurry trays underneath the slatted floors were discontinuously discharged in the middle and at the end of each fattening period. There were eight pens to a row on both sides of the two alleys (Fig 1). Two pens near the entrances of each barn were used for separating pigs owing to various health issues. The barns were stocked from farrowing operations on the same farm. At placing, animals were aged approximately eleven weeks and weighed on average 31 kg each. They remained in their barn for fourteen to sixteen weeks until marketable body weight (117–127 kg) was reached. Stocking occurred at four weeks intervals starting with barn 2 followed by barn 1, barn 3 and barn 4. Each barn was cleaned and disinfected before restocking. Drinking water was provided by nipple drinkers and pigs were fed an industry-standard diet provided by an automated wet feeding system. The piglets were vaccinated against *M*. *hyopneumoniae* and porcine circovirus 2.

General measures of biosecurity were disinfection of car tires at the entrance of the farm area, proprietary overalls and boots for employees and visitors for each facility and disinfection of boots before entering each barn. All visitors entering the barns must confirm by signature that they had no contact with pigs from outside the facility for the last 48 h.

### Ventilation systems at the test facility

Three barns (barn 1, 2, and 4) were equipped with air filtration and the remaining barn (barn 3) was used as a reference without any air filtration. Technical information of the different air filter types can be taken from S1 Table. Each barn had twelve exhaust (negative pressure) fans (Fig 1) which can be adjusted to provide adequate ventilation rates.

#### Barn 1 (supply air filter modules)

**Fig 2:**
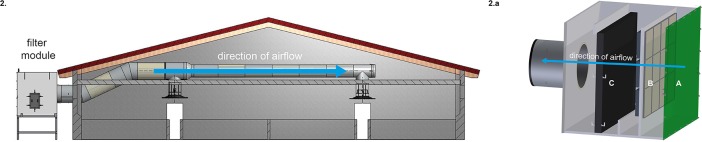
Distribution of fresh air into barn 1 and structure of the supply air filter modules of barn 1. (2.) This figure exemplarily illustrates the air influx for one supply air filter module. Five of these modules were installed at the left long side of barn 1. Each module was connected to a corresponding ventilation pipe along with two air distributors. The direction of airflow is indicated by a blue arrow. (2.a) Supply air filter modules were composed of: A—windshield, B—prefilters and secondary compact filters, C—adiabatic cooling device. Prefilter dimensions were 592 mm (length) x 592 mm (height) x 48 mm (width). Secondary compact filters sized 592 mm x 592 mm x 292 mm (see S1 Table). The direction of airflow is indicated by a blue arrow.

Barn 1 was equipped with five supply air filter modules (high-velocity ventilation) installed at the left long side of the barn. From each module fresh air was delivered into the barn by a ventilation pipe and two air distributors (Fig 2). Each module was composed of a windscreen, twelve prefilters (MERV 6–8) and twelve secondary filters (MERV 16) and an adiabatic cooling device for optional use (Fig 2A). The maximum volume flow rate of each module was 20,000 m^3^/h. Fresh air inlets in the walls which had been used for fresh air delivery before were tightly sealed.

#### Barn 2 (supply air filter attic)

**Fig 3:**
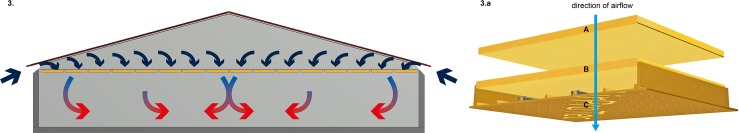
Cross section of barn 2 demonstrating the distribution of fresh air and composition of the perforated polyurethane cassettes used for supply air filtration in barn 2. (3.) Each filter unit consisted of a polyurethane cassette and two glass wool filters. These units (indicated in yellow) were installed into the barn attic without gaps. Fresh air entered the attic via slots underneath the roof (dark blue arrows). Air flow (indicated as multicolored arrows) was regulated by negative pressure. (3.a) Each air filter unit in the attic of barn 2 was composed of two glass wool filter mats (A, B) embedded in a perforated polyurethane cassette (C). Each mat sized 1,200 mm x 1,200 mm and had a thickness of 40 mm. The direction of airflow through each cassette is indicated by a blue arrow.

A ceiling consisting of perforated polyurethane cassettes filled with two layers of a specifically treated glass wool filter (MERV 14–16) was installed into the attic of barn 2 (Fig 3 and 3A). More than 340 of these cassettes served as a diffuse fresh air supply system (displacement ventilation method). The maximum volume flow rate was 290 m^3^/h for each cassette. As described for barn 1, fresh air inlets in the walls (Fig 1) which had been used for fresh air delivery before were tightly sealed.

#### Barn 3 (without air filtration system)

Barn 3 as our reference without any air filtration was equipped with fresh air inlets on both longitudinal sides (Fig 1). Air flow was regulated by negative pressure.

#### Barn 4 (recirculating air filtration modules)

**Fig 4:**
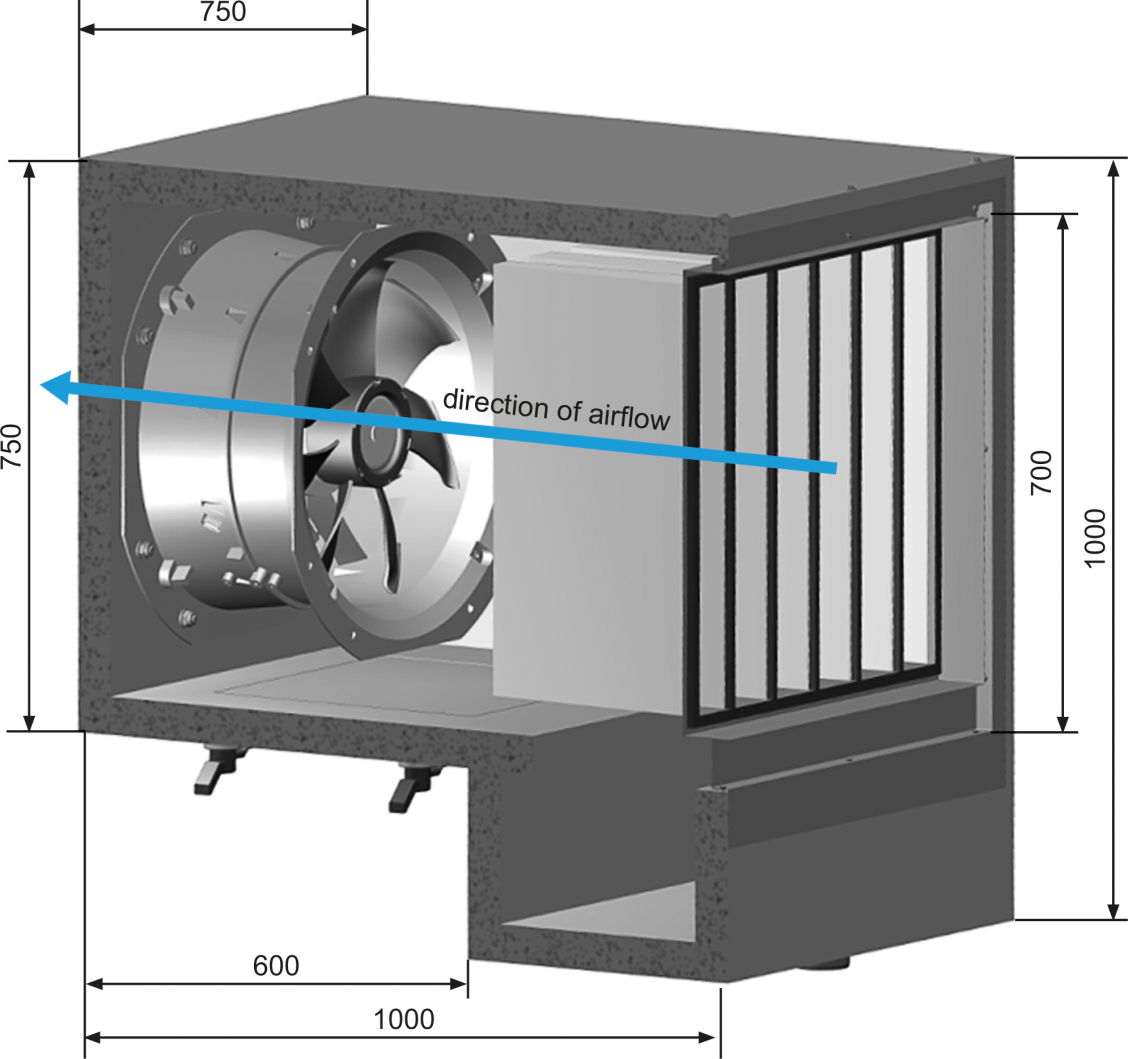
Cross section of a recirculating air filter module from barn 4. Each recirculating air filter module consisted of a plastic housing equipped with a pocket air filter and a fan. The air volume flow rate was regulated via the fan. Module dimensions (mm) are specified and the direction of airflow is indicated by a blue arrow. Barn 4 was equipped with four of these modules.

Fresh air was delivered into barn 4 by fresh air inlets on both longitudinal sides of the barn (Fig 1). In addition, four recirculating air filtration modules with a flow rate of 3,000 m^3^/h were installed indoors (indicated by a framed X in Fig 1) assuming an air exchange rate of 3x stable volume/h. These were initially constructed as plastic housings with an integrated vibrating dust sieve and an axial fan. However, dust particles remained stuck on the sieve and were not removed by the vibrating function as intended. Therefore, the sieve was removed and the recirculating air filter modules were equipped with a pocket air filter (MERV 5–6; Fig 4) instead. This was done during the first fattening period. Pocket air filter replacement was necessary once during each fattening period for these recirculating air filtration modules.

### Evaluation of indoor air quality

Investigations were done at two week intervals. Barn 1 and 2 were sampled on the same day as were barn 3 and 4. On every sampling day, all measurements were conducted between 9:00 a.m. and 12:00 p.m.. Airborne bacteria, total dust particles, and ammonia were measured one day before the pigs arrived as a “base line”. Temperature (indoor and outdoor), relative humidity (DOL 114 humidity and temperature sensor, dol sensors, Roslev, Denmark), ventilation flow rate (measuring fan integrated into exhaust air ducts, REVENTA^®^ GmbH, Horstmar, Germany), and carbon dioxide (CO_2_; OPN-CO_2_-E2 sensor, Hotraco Agri, AC Hegelsom, The Netherlands) were continuously recorded with the following exception: During the first fattening period, and in barn 2 also during the second fattening period, CO_2_ was only measured at two week intervals with a Testo 535-CO_2_ meter (Testo SE & Co. KGaA, Lenzkirch, Germany). In addition, ammonia (NH_3_) concentration was measured every two weeks by means of a portable NH_3_ Gas Detector (Model CMS, Dräger Safety AG & Co. KGaA, Lübeck, Germany). All devices were calibrated once a year by the manufacturer.

Wet cyclone technology (Coriolis®μ Air sampler, Bertin Technologies, Montigny le Bretonneux, France) was used to sample airborne bacteria [23]. Sampling was carried out at pig level for 10 minutes with a volume flow rate of 300 l air/min (i.e. total volume of 3,000 l) at six standard sampling positions per barn (Fig 1). Air samples were collected in Coriolis®μ cones filled with 10 ml phosphate buffered saline (PBS) supplemented with 0.005% Tween 80 (Carl Roth GmbH + Co. KG, Karlsruhe, Germany). Samples were transported on ice to the laboratory and analyses started at the day of sampling. Dust sampling was carried out with the DustTrak™ DRX Aerosol Monitor 8533 (TSI GmbH, Aachen, Germany) for 10 minutes each at one sampling point per alley (Fig 1) randomly selected out of six sampling points per barn. This procedure was based on the results of a series of preliminary measurements where no differences in mean dust values collected at various different sampling points were found. The DustTrak™ DRX Aerosol Monitor simultaneously measures size-segregated mass fraction concentrations corresponding to particles ≤1 μm, ≤2.5 μm, respirable particles, ≤10 μm, and total particle size fractions. Calibration was done yearly by the manufacturer. Generally, no measurements were done during feeding and slurry discharge.

### Laboratory analyses of air samples

Coriolis®μ cones (Bertin Technologies) were thoroughly vortexed after sampling and analyzed. Two 1.5 ml aliquots were stored at -80°C as a backup until further use. Bacteria were enumerated by the spread-plate method. Each sample was serially diluted 1:10 in tryptic soy broth (Carl Roth GmbH + Co. KG) and was plated in a volume of 0.1 ml onto Columbia sheep blood agar (CSA; Oxoid, Wesel, Germany) and MRSA Chromagar (MAST Diagnostica GmbH, Reinfeld, Germany) in duplicates. For the detection of *Enterobacteriacae* an undiluted 0.1 ml aliquot was taken from each sample and plated onto Brilliance^TM^ *Escherichia* (*E*.) *coli*/coliform selective agar (Oxoid) in duplicates. Plates were incubated aerobically for 48 h at 37°C. After the first 24 h CSA plates were further incubated at 37°C using 5% CO_2_. After 48 h bacteria colonies were counted and the amount of colony forming units (CFU) was calculated per 1 m^3^ air. Colonies suggestive of MRSA on MRSA Chromagar were sub-cultured and from each sub-culture an inoculation loop of colony material was diluted in 200 μl PBS, heated for 10 min at 99°C and stored at -20°C until further use. MRSA was confirmed by two conventional PCRs targeting the *mecA* and the *spa* gene according to Strommenger et al. [24] and Harmsen et al. [25] with minor modifications.

Amplification products were UV-analyzed on a 2% agarose gel dyed with HD Green™ Plus (Intas Science Imaging Instruments GmbH, Goettingen, Germany). In addition, all air samples were examined by real-time RT-PCR to detect PRRSV. RNA extraction was carried out with the QIAamp® RNA Mini Kit (Qiagen, Hilden, Germany) as recommended by the manufacturer. PRRSV detection was carried out using the SuperScript III Platinum® One-Step Quantitative RT-PCR System (Invitrogen, Carlsbad, USA) according to Dee et al. [26] with minor modifications. Real-time RT-PCR was performed on the Mx3000p platform (Stratagene, La Jolla, USA) with following cycling conditions of: 50°C for 15 min (reverse transcription), 95°C for 2 min, followed by 40 cycles of denaturation at 95°C for 15 sec and annealing at 60°C for 30 sec. The limit of detection (95% probability) of this quantitative real-time RT-PCR was 6 copies/μl.

### Animal sampling and pig performance

Blood serum samples were taken from fifteen randomly selected pigs per barn five days after arrival by the farm veterinarian. The study was approved by the ethics committee of the veterinary faculty of the University of Leipzig. Procedures fully complied with the German Protection of Animals Act and were notified by the Landesdirektion Sachsen (reference number TVV A09/15). Animals were ear tagged and a second blood sample was taken at the end of the fattening period. All blood serum samples were tested for antibodies against PRRSV (ELISA), Influenza A viruses H1N1 and H3N2 (hemagglutination inhibition assay), and *Actinobacillus pleuropneumoniae* (APP; ELISA) at the Landesuntersuchungsanstalt für das Gesundheits- und Veterinärwesen in Saxony, Dresden, Germany. At slaughter, carcasses were examined with respect to respiratory diseases by the Schweinegesundheitsdienst (SGD) Saxony. Findings were summarized in a scoring system [27–30] including the occurrence of pneumonia, pleurisy and pericarditis (Table 1). In addition, pig performance was evaluated which included the factors average daily weight gain, mortality [31] and animal treatment [32]. The animal treatment was recorded as animal treatment index (ATI) which was calculated by the following equation: (number of animals treated x number of treatment days)/numbers of animals in the barn [32]. Data for pig performance were obtained from inventory data and were also summarized in a score system (Table 2). Animal health was monitored every second week with the help of a checklist with particular attention to respiratory symptoms. Sneezing and coughing was counted twice for 10 min per stable and was evaluated with a score system (Table 3).

**Table 1:** Parameters used to score lung health at slaughter.

Parameters	Score according to occurrence			
	0	1	2	3
Pneumonia	<1%	1–10%	11–30%	≥30%
Pleurisy	<1%	1–10%	11–30%	≥30%
Pericarditis	<1%	>1%		
**Maximum score**				**7**

Lung health score modified according to Richter [29]: 0–1 = very good, 2–3 = good, 4–5 = medium, 6–7 = bad.

**Table 2:** Pig performance index.

Parameters	Score			
	0	1	2	3
Daily gain (g)	>850	>800–850	>750–800	700–750
Mortality (%)	<2	2- <3	3–4	>4
ATI	<10	11–20	21–40	>40
**Maximum score**				**9**

Scoring was performed according to [28–30] and pig performance was evaluated following Richter [29]: 0 = very good, 1–3 = good, 4–6 = medium, 7–9 = bad.

**Table 3:** Index of clinical signs to evaluate lung health.

Clinical signs	Score		
	0	2	4
Sneezing	< 5%	≤ 5% ≥ 30%	> 30%
Coughing	< 5%	≤ 5% ≥ 30%	> 30%
**Maximum Score**			**8**

Scoring was performed according to Richter [29] and Brauer [30] and lung health was assessed according to Richter [29] and Dickhaus [28]: 0 = good, 2–4 = medium, 6–8 = bad.

### Pathogen load inside the filters after one year in use

One year after implementation of the filter systems samples were taken from the filter matter of barn 1 and 2 and were investigated for total aerobic germ load and the presence of PRRSV. One prefilter and one secondary filter of each module were examined. Five samples (approximately 1 cm x 1 cm each) of each prefilter were pooled. The secondary filter was sampled accordingly. From the filter mat in barn 2 a piece of 2 cm x 2 cm was cut out in the center of the attic. Pooled samples of each filter were rinsed for 10 min in tryptic soy broth and the filter material was discarded thereafter. The total bacteria amount was enumerated by the spread-plate method as described above. In addition, RNA extraction and PRRSV quantitative real-time RT-PCR was carried out as described above.

### Statistical analyses

Statistical analysis was made with SPSS Statistic 22 (IBM Deutschland GmbH, Ehningen, Germany). For comparison of the four barns regarding airborne bacteria, CO_2_- and NH_3_-concentration Kruskal-Wallis-Tests were used. The significance level was p ≤ 0.05 (two-sided). At all points in time animal numbers varied in the four barns due to restocking at four week intervals and successive transportation of pigs to the slaughterhouse. In order to make results comparable, only data collected at a stock of ≥800 animals were included in the analyses.

## Results

### Air quality

#### Bacterial load in air samples

The “base line” (i.e. after cleaning and disinfection and one day before restocking) revealed total bacteria counts of 60 cfu/m^3^ (barn 4) to 2x10^4^ cfu/m^3^ (barn 1). Most of these “base line” values were >10^3^ cfu/m^3^ (Tables 4–7). Bacterial counts continuously increased after restocking in all barns with a few exceptions (Tables 4–7). Two weeks after the arrival of the piglets total bacteria counts were mostly higher than 10^5^ cfu/m^3^. In most barns the total amount of bacteria exceeded 10^6^ cfu/m^3^ at least once during the fattening period. Regarding the total amount of bacteria there were no significant differences (p = 0.824) between barns with and without air filtration. The amount of airborne MRSA in the cleaned and disinfected barns (“base line”) after cleaning and disinfection ranged from zero to 36 cfu/m^3^. Within two weeks after restocking airborne MRSA increased by more than 10,000-times (Table 6; barn 4, first fattening period). Maximum MRSA/m^3^ was 7.8x10^4^ cfu (Table 7; barn 3, second fattening period). In contrast to total airborne bacteria and MRSA, *E*. *coli* and coliform bacteria were detected irregularly. “Base line” values were mainly zero for these bacteria with the exception of samples taken before fattening period 3 in barn 1 (34 cfu of *E*. *coli*/m^3^, coliform bacteria 95 cfu/m^3^). Overall, coliform bacteria varied between 0 cfu/m^3^ and 458 cfu/m^3^, and *E*. *coli* ranged from 0 cfu/m^3^ to 361 cfu/m^3^ (Tables 4–7). PRRSV was not detected in any air sample by real-time RT-PCR.

**Table 4:** Airborne bacteria and dust concentration in barn 1 equipped with supply air filter modules.

FP	Season	Week	No. of animals	Ventilation flow (m^3^/h ± SD)	Dust total (mg/m^3^ ± SD)	Total bacteria (cfu/m^3^ ± SD)	MRSA (cfu/m^3^ ± SD)	Coliform bacteria (cfu/m^3^ ± SD)	*E*. *coli* (cfu/m^3^ ± SD)
1	Summer	0	0	na	0.002 ± 0.001	178 ± 59	22 ± 54	0	0
		2	952	16,688 ± 745	0.059 ± 0,017	142,833 ± 77,176	9,765 ± 6,608	4 ± 7	1 ± 3
		4	951	17,682 ± 1,102	0.072 ± 0.018	314,833 ± 74,593	17,300 ± 9,496	1 ± 3	30 ± 63
	Autumn	6	945	20,911 ± 3,792	0.127 ± 0.036	492,667 ± 209,658	26,100 ± 7,395	0	0
		8	941	16,937 ± 1,102	0.160 ± 0.110	870,667 ± 407,847	52,700 ± 33,547	0	1 ± 3
		10	939	16,937 ± 1,102	0.200 ± 0.098	951,167 ± 352,255	44,517 ± 17,049	2 ± 5	12 ± 29
		12	876	15,073 ± 9,605	0.135 ± 0.033	1,592,833 ± 516,713	31,457 ± 15,888	0	0
2	Winter	0	0	na	0.043± 0.006	789 ± 342	6 ± 8	0	0
		2	na	na	na	na	na	na	na
		4	945	15,620 ± 754	0.172 ± 0.071	693,500 ± 96,874	33,100 ± 31,961	0	6 ± 9
		6	944	18,303 ± 641	0.189 ± 0.055	706,000 ± 397,394	19,700 ± 9,758	0	0
		8	940	17,309 ± 1,183	0.135 ± 0.035	847,000 ± 206,062	17,910 ± 6,883	48 ± 107	18 ± 28
		10	938	18,303 ± 849	0.242 ± 0.143	1,528,333 ± 670,861	27,000 ± 17,164	2 ± 4	8 ± 12
		12	802	13,831 ± 625	0.184 ± 0.011	1,242,667 ± 894,841	4,527 ± 2,590	27 ± 50	27 ± 42
3	Spring	0	0	na	0.009 ± 0	19,906 ± 48,543	2 ± 4	95 ± 233	34 ± 84
		2	956	12,838 ± 1,463	0.181 ± 0.074	221,000 ± 65,097	11,030 ± 8,228	2 ± 5	9 ± 23
		4	952	19,794 ± 1,924	0.114 ± 0.034	183,333 ± 78,237	10,048 ± 6,838	2 ± 4	5 ± 12
		6	951	15,322 ± 811	0.188 ± 0.008	800,000 ± 451,541	17,100 ± 6,492	0	3 ± 8
		8	943	32,712 ± 248	0.072 ± 0.023	2,029,500 ± 1,726,851	59,900 ± 56,756	6 ± 7	24 ± 58
		10	938	26,750 ± 1,307	0.071 ± 0.021	1,049,333 ± 545,611	25,485 ± 11,231	2 ± 5	9 ± 8
	Summer	12	895	36,190 ± 1,307	0.092 ± 0.073	2,073,500 ± 1,949,409	18,993 ± 14,426	2 ± 5	10 ± 24

Measurements were taken at two week intervals and sampling was performed between 9 a.m. and 12 p.m.. The ventilation flow rates represent the mean ± standard deviation (SD) of ventilation data recorded during sampling time. Total dust values (mean ± SD) were calculated from data collected by the DustTrak™ DRX Aerosol Monitor over 10 min at two sampling points. Total dust comprises particles ≤1 μm, ≤2.5 μm, respirable particles, and ≤10 μm. The amount of total bacteria, MRSA, coliform bacteria, and *E*. *coli* represents the arithmetic mean ± SD of measurements done at six sampling points. Seasons were defined according to the astronomical calendar: spring (21^st^ March to 20^th^ June), summer (21^st^ June to 22^nd^ September), autumn (23^rd^ September to 21^st^ December), winter (22^nd^ December to 20^th^ March). FP—fattening period; na—data not available

**Table 5:** Airborne bacteria and dust concentration in barn 2 equipped with a supply air filter attic.

FP	Season	Week	No. of animals	Ventilation flow (m^3^/h ± SD)	Dust total (mg/m^3^ ± SD)	Total bacteria (cfu/m^3^ ± SD)	MRSA (cfu/m^3^ ± SD)	Coliform bacteria (cfu/m^3^ ± SD)	*E*. *coli* (cfu/m^3^ ± SD)
1	Summer	0	0	na	0.003 ± 0.004	207 ± 98	3 ± 7	0	0
		2	954	34,978 ± 4,330	0.021 ± 0.004	29,419 ± 14,297	4,188 ± 2,809	0	0
		4	952	38,383 ± 1,589	0.043 ± 0.019	179,167 ± 133,333	10,158 ± 6,630	0	4 ± 10
		6	945	18,176 ± 1,251	0.140 ± 0.052	551,667 ± 197,526	66,517 ± 48,101	0	4 ± 5
		8	934	25,214 ± 936	0.105 ± 0.064	824,500 ± 285,868	43,050 ± 23,448	3 ± 4	3 ± 4
	Autumn	10	928	24,397 ± 4,395	0.142 ± 0.064	833,833 ±317,363	30,650 ± 12,225	1 ± 3	0
		12	912	21,581 ± 3,176	0.131 ± 0.064	2,296,500 ± 4,166,893	39,250 ± 24,197	0	125 ± 306
2	Autumn	0	0	na	na	na	na	na	na
		2	960	15,542 ± 1,854	0.162 ± 0.001	351,500 ± 163,342	45,600 ± 32,810	0	0
		4	954	15,315 ± 1,529	0.643 ± 0.689	620,500 ± 471,640	26,235 ±30,498	47 ± 66	12 ± 16
		6	na	na	na	na	na	na	n.a
	Winter	8	945	16,450 ± 1,304	0.099 ± 0.01	1,047,500 ± 17,678	29,450 ± 4,596	18 ± 9	6 ± 9
		10	942	19,856 ± 945	0.216 ± 0.047	1,385,000 ± 63,640	20,650 ± 14,354	0	0
		12	891	18,039 ± 1,979	0.083 ± 0.039	1,153,667 ± 658,242	17,983 ± 12,706	2 ± 5	2 ± 5
3	Spring	0	0	na	0.145 ± 0.002	786 ± 1,111	6 ± 10	0	0
		2	944	15,996 ± 642	0.241 ± 0.217	291,000 ± 46,463	10,932 ± 6,638	2 ± 5	0
		4	942	18,721 ± 435	0.073 ± 0.004	4,364,667 ± 9,669,106	29,050 ± 16,342	3 ± 8	16 ± 38
		6	939	20,083 ± 1,940	0.084 ± 0.01	880,833 ± 550,363	15,745 ± 13,037	30 ± 73	0
		8	936	26,440 ± 3,327	0.059 ± 0.013	646,333 ± 315,909	6,153 ± 3,034	0	2 ± 4
		10	933	19,629 ± 642	0.146 ± 0.079	1,513,833 ± 891,356	14,992 ± 19,999	4 ± 6	17 ± 15
		12	847	36,431 ± 0	0.082 ± 0.032	1,128,167 ± 353,883	7,878 ± 5,549	13 ± 17	43 ± 44

Measurements were taken at two week intervals and sampling was performed between 9 a.m. and 12 p.m.. The ventilation flow rates represent the mean ± standard deviation (SD) of ventilation data recorded during sampling time. Total dust values (mean ± SD) were calculated from data collected by the DustTrak™ DRX Aerosol Monitor over 10 min at two sampling points. Total dust comprises particles ≤1 μm, ≤2.5 μm, respirable particles, and ≤10 μm. The amount of total bacteria, MRSA, coliform bacteria, and *E*. *coli* represents the arithmetic mean ± SD of measurements done at six sampling points. Seasons were defined according to the astronomical calendar: spring (21^st^ March to 20^th^ June), summer (21^st^ June to 22^nd^ September), autumn (23^rd^ September to 21^st^ December), winter (22^nd^ December to 20^th^ March). FP—fattening period; na—data not available

**Table 6:** Airborne bacteria and dust concentration in barn 4 equipped with recirculating air filtration modules.

FP	Season	Week	No of animals	Ventilation flow (m^3^/h ± SD)	Dust total (mg/m^3^ ± SD)	Total bacteria(cfu/m^3^ ± SD)	MRSA (cfu/m^3^ ± SD)	Coliform bacteria (cfu/m^3^ ± SD)	*E*. *coli* (cfu/m^3^ ± SD)
1	Autumn	0	0	na	0.101 ± 0.01	1,584 ± 3,192	0	0	0
		2	960	12,976*	0.158 ± 0.025	197,000 ±37,942	14,710 ± 10,248	4 ± 6	6 ± 14
		4	960	17,707 ± 1,745	0.073 ± 0.025	239,283 ± 115,440	10,217 ± 3,948	12 ± 20	8 ± 10
		6	944	17,594 ± 999	0.132 ± 0.023	1,110,167 ± 662,986	29,495 ±12,723	0	10 ± 19
		8	943	17,144 ± 1,489	0.280 ± 0.084	1,676,667 ± 504050	50,600 ± 27,853	29 ± 60	21 ± 51
		10	na	na	na	na	na	na	na
	Winter	12	832	16,581 ± 1,185	0.183 ± 0.06	2,071,667 ± 3,014,239	32,000 ± 23,064	6 ± 7	0
2	Winter	0	0	na	0.041 ± 0.013	8,022 ± 16,172	35 ± 53	0	0
		2	939	14,553 ± 863	0.095 ± 0.034	1,293,500 ± 750,001	53,100 ± 20,631	292 ± 638	361 ± 688
		4	934	20,185*	0.209 ± 0.076	365,167 ± 180,770	27,150 ± 11,916	7 ± 8	7 ± 16
	Spring	6	931	16,806 ± 260	0.112 ± 0.018	336,333 ± 174,240	11,637 ± 4,948	5 ± 13	0
		8	928	24,917 ± 1,073	0.226 ± 0.085	609,500 ± 534,188	21,683 ± 10,912	8 ± 13	0
		10	926	17,707 ± 1,192	0.188 ± 0.057	888,833 ± 384,566	19,383 ±14,055	0	2 ± 4
		12	832	20,321 ± 3,848	0.135 ± 0.008	846,667 ± 440,503	9,290 ± 8,267	0	2 ± 4
3	Spring	0	0	na	0.076 ± 0.003	60 ± 16	0	0	0
		2	956	18,969 ± 1,185	0.054 ± 0.006	120,067 ± 68,951	4,128 ± 2,290	6 ± 6	4 ± 6
	Summer	4	950	24,917± 2,341	0.089 ± 0.042	477,250 ± 460,130	2,330 ± 16,594	83 ± 2	0
		6	948	40,012 ± 0	0.051 ± 0.019	257,000 ± 183,715	17,620 ± 12,952	0	0
		8	942	36,633 ± 1,352	0.068 ± 0.004	650,000 ± 248,988	46,210 ± 64,956	10 ± 24	0
		10	931	26,854 ± 2,506	0.051 ± 0.018	690,667 ± 460,752	32,400 ± 18,463	0	10 ± 13
		12	916	35,416 ± 225	0.061 ± 0.018	2,034,667 ± 2,022,235	28,625 ± 31,062	0	13 ± 16

Measurements were taken at two week intervals and sampling was performed between 9 a.m. and 12 p.m.. The ventilation flow rates represent the mean ± standard deviation (SD) of ventilation data recorded during sampling time. Total dust values (mean ± SD) were calculated from data collected by the DustTrak™ DRX Aerosol Monitor over 10 min at two sampling points. Total dust comprises particles ≤1 μm, ≤2.5 μm, respirable particles, and ≤10 μm. The amount of total bacteria, MRSA, coliform bacteria, and *E*. *coli* represents the arithmetic mean ± SD of measurements done at six sampling points. Seasons were defined according to the astronomical calendar: spring (21^st^ March to 20^th^ June), summer (21^st^ June to 22^nd^ September), autumn (23^rd^ September to 21^st^ December), winter (22^nd^ December to 20^th^ March). FP—fattening period; na—data not available

*data logging over time by the computer system failed and the given value represents a single value taken directly from the control panel in front of the barn

**Table 7:** Airborne bacteria and dust concentration in barn 3 without air filtration.

FP	Season	Week	No of animals	Ventilation flow (m^3^/h ± SD)	Dust total (mg/m^3^ ± SD)	Total bacteria (cfu/m^3^ ± SD)	MRSA (cfu/m^3^ ± SD)	Coliform bacteria (cfu/m^3^ ± SD)	*E*. *coli* (cfu/m^3^ ± SD)
1	Autumn	0	0	na	0.019 ± 0.01	1,357 ± 1,133	36 ± 56	0	0
		2	943	22,145 ± 1,398	0.117 ± 0.039	52,750 ± 26,505	4,347 ± 2,318	6 ± 7	0
		4	940	16,644 ± 889	0.136 ± 0.014	248,383 ± 161,838	12,538 ± 12,890	1 ± 3	0
		6	938	24,433*	0.242 ± 0.126	963,000 ± 681,964	37,533 ± 35,775	0	8 ± 14
		8	931	25,309 ± 1,838	0.122 ± 0.021	854,500 ± 276,755	23,283 ± 8,609	130 ± 308	52 ± 95
		10	916	23,119 ± 2,677	0.174 ± 0.055	1,443,667 ± 625,724	47,217 ± 19,015	2 ± 5	0
		12	861	16,888 ± 1,159	0.243 ± 0.078	2,555,000 ± 702,446	51,400 ± 24,626	19 ± 28	13 ±21
2	Winter	0	0	na	0.025 ± 0.004	1,535 ± 665	36 ± 35	0	0
		2	941	17,374 ± 487	0.130 ± 0.011	397,500 ± 323,539	65,333 ± 27,735	2 ± 4	12 ± 19
		4	936	15,573 ± 243	0.158 ± 0.103	363,333 ± 112,708	37,117 ± 14,687	5 ± 8	0
		6	928	15,330 ± 466	0.135 ± 0.018	1,032,000 ± 410,100	77,550 ± 21,642	35 ± 81	9 ± 12
		8	926	15,671*	0.119 ± 0.002	829,833 ± 388,189	48,333 ± 34,678	0	2 ± 5
	Spring	10	924	17,277 ± 613	0.140 ± 0.002	706,400 ± 501,855	20,475 ± 10,978	4 ± 9	0
		12	894	27,597 ± 1,225	0.208 ± 0.052	990,333 ± 470,590	33,752 ± 21,722	3 ± 8	0
3	Spring	0	600^#^	na	0.121 ± 0.05	8,500 ± 6,907	58 ± 70	2 ± 5	0
		2	953	15,305 ± 466	0.123 ± 0.045	351,900 ± 428,667	30,020 ± 53,118	7 ± 13	78 ± 181
		4	950	25,285 ± 1,004	0.093 ± 0.01	164,383 ± 111,485	17,435 ± 16,423	196 ± 476	0
		6	948	26,624 ± 1,288	0.093 ± 0.049	393,833 ± 240,655	36,467 ± 28,869	60 ± 142	8 ± 6
	Summer	8	945	31,492 ± 3,192	0.081 ±0	748,333 ± 352,408	30,317 ± 14,559	2 ± 5	2 ± 5
		10	938	40,985 ± 0	0.058 ± 0.004	323,833 ± 105,511	11,232 ± 7,263	83 ± 2	0
		12	932	39,037 ± 0	0.086 ± 0.008	993,667 ± 981,515	8,887 ± 4,115	458 ± 1123	79 ± 194

Measurements were taken at two week intervals and sampling was performed between 9 a.m. and 12 p.m.. The ventilation flow rates represent the mean ± standard deviation (SD) of ventilation data recorded during sampling time. Total dust values (mean ± SD) were calculated from data collected by the DustTrak™ DRX Aerosol Monitor over 10 min at two sampling points. Total dust comprises particles ≤1 μm, ≤2.5 μm, respirable particles, and ≤10 μm. The amount of total bacteria, MRSA, coliform bacteria, and *E*. *coli* represents the arithmetic mean ± SD of measurements done at six sampling points. Seasons were defined according to the astronomical calendar: spring (21^st^ March to 20^th^ June), summer (21^st^ June to 22^nd^ September), autumn (23^rd^ September to 21^st^ December), winter (22^nd^ December to 20^th^ March). FP—fattening period; na—data not available

*data logging over time by the computer system failed and the given value represents a single value taken directly from the control panel in front of the barn

^#^due to organizational reasons sampling at the abandoned barn was not possible

#### Total dust concentration

Dust concentration was measured on two randomly chosen spots per barn. The total dust concentration ranged from 0.059 to 0.242 mg/m^3^ (barn 1), 0.021 to 0.643 mg/m^3^ (barn 2), 0.058 to 0.243 mg/m^3^ (barn 3), and 0.051 to 0.226 mg/m^3^ (barn 4), respectively (Tables 4–7). On average, barn 4 with recirculating air filtration revealed the lowest total dust concentration (0.12 mg/m^3^). Barn 2 (supply air filter attic) and barn 1 (supply air filter modules) achieved a mean total dust concentration of 0.14 mg/m^3^. The control (barn 3) revealed a similar result (0.13 mg/m^3^).

#### CO_2_- and NH_3_-concentration

According to the statutory requirements regarding pig husbandry conditions in Germany the critical value of CO_2_ is set at 3,000 ppm [33]. Most common, the level of CO_2_ was less than the critical value, depending on the ventilation flow rate and outdoor temperature (S2–S5 Tables). During the first fatting period CO_2_ was measured with a handheld Testo 535 device. Most values exceeding the critical value were determined with this handheld device. Overall, the concentration varied between 1,130 ppm (during summer) and 4,363 ppm (during autumn). The latter value corresponded with the lowest outdoor temperature of 1.4°C. Among all barns, barn 2 revealed the highest CO_2_-concentration especially in autumn and winter of the second fattening period. CO_2_-concentration did not differ between barns with and without filtration (p = 0.296). Ammonia was measured every two weeks also using a handheld device. According to the German Tierschutz-Nutztierhaltungsverordnung [33], the threshold limit value for ammonia is set at 20 ppm. The concentrations measured during our study period ranged from 8.65 ppm to 31.62 ppm (S2–S5 Tables). Highest values were reported during autumn and winter and were generally highest (25.96 ppm) in barn 2 with the filter attic. The lowest concentrations of ammonia were seen in barn 4 with the recirculating air filtration (17.37 ppm on average). However, there were no significant differences of ammonia concentration when comparing filtered and non-filtered barns (p = 0.184).

#### Temperature and relative humidity

Indoor temperature varied from 22.7°C to 31.7°C with a mean of 25.0°C. The outdoor temperature ranged from 1.4°C to 29.4°C during data collection at the facility. Relative humidity indoors varied from 62% to 80% (S2–S5 Tables) but mostly averaged between 67% and 75%. There were no obvious differences between the filtered and non-filtered barns. Unfortunately, measurements of the relative humidity were unfeasible during the first fattening period in all barns and also during half of fattening period 2 in barn 2.

### Animal health and pig performance

The results obtained are presented in Table 8 (for pig performance scores see Table 2). There was no difference in daily weight gain and ATI, which were continuously defined by a score of 0 (i.e. very good). In contrast, the mortality varied during the different fattening periods and between barns and mostly influenced pig performance. The lowest mortality rate (2% on average) was recorded in animals reared in barn 1 with the supply air filter module. In all barns deceased animals predominantly revealed intestinal disorders. Nevertheless, pig performance in all barns was evaluated as very good to good (i.e. score 0–3). Referring to the index of respiratory signs (i.e. the amount auf sneezing and coughing) animals from barn 4 (with recirculating air filtration) revealed good lung health during two of the three fattening periods and medium lung health in the third period. Animals from barn 1–3 (Table 9) showed good lung health in the first fattening period and medium lung health in the remaining two fattening periods.

**Table 8:** Pig performance parameters obtained from three consecutive fattening periods.

Performance	Barn 1: Supply air filter modules						Barn 2: Supply air filter attic						Barn 4: Recirculating air filtration modules						Barn 3: Without filtration					
	1. FP		2. FP		3. FP		1. FP		2. FP		3. FP		1. FP		2. FP		3. FP		1. FP		2. FP		3. FP	
	value	S	value	S	value	S	value	S	value	S	value	S	value	S	value	S	value	S	value	S	value	S	value	S
Daily gain (g)	916	0	922	0	921	0	951	0	976	0	885	0	960	0	900	0	930	0	965	0	944	0	923	0
Mortality (%)	1.5	0	2.1	1	2.4	1	4.5	3	1.98	0	3.4	2	3.5	2	1.9	0	4.4	3	3.3	2	1.98	0	3.5	2
ATI	7.1	0	0.1	0	0	0	0.2	0	0.3	0	7.5	0	7.0	0	7.1	0	0	0	8.1	0	0.1	0	0.1	0
Total score	0		1		1		3		0		2		2		0		3		2		0		2	

Evaluation of pig performance according to Richter [29]: 0 = very good, 1–3 = good, 4–6 = medium, 7–9 = bad; FP—fattening period; S—score

**Table 9:** Respiratory health in pigs kept in barns with and without air filtration.

Clinical signs	Barn 1: Supply air filter modules						Barn 2: Supply air filter attic						Barn 4: Recirculating air filtration modules						Barn 3: Without filtration					
	1. FP		2. FP		3. FP		1. FP		2. FP		3. FP		1. FP		2. FP		3. FP		1. FP		2. FP		3. FP	
	OCC	S	OCC	S	OCC	S	OCC	S	OCC	S	OCC	S	OCC	S	OCC	S	OCC	S	OCC	S	OCC	S	OCC	S
Sneezing (%)	2.5	0	6.2	2	5.4	2	1.5	0	6.2	2	5.0	2	4.6	0	3.9	0	5.8	2	3.5	0	5.2	2	5.1	2
Coughing (%)	0.1	0	0.1	0	0.3	0	1.3	0	1.2	0	1.0	0	0.2	0	0.1	0	0.1	0	0.2	0	0.2	0	0.5	0
Total score	0		2		2		0		2		2		0		0		2		0		2		2	

Assessment of lung health according to Richter [29] and Dickhaus [28]: 0 = good, 2–4 = medium, 6–8 = bad; FP—fattening period; OCC—occurrence; S—score

#### Seroprevalence

**Fig 5:**
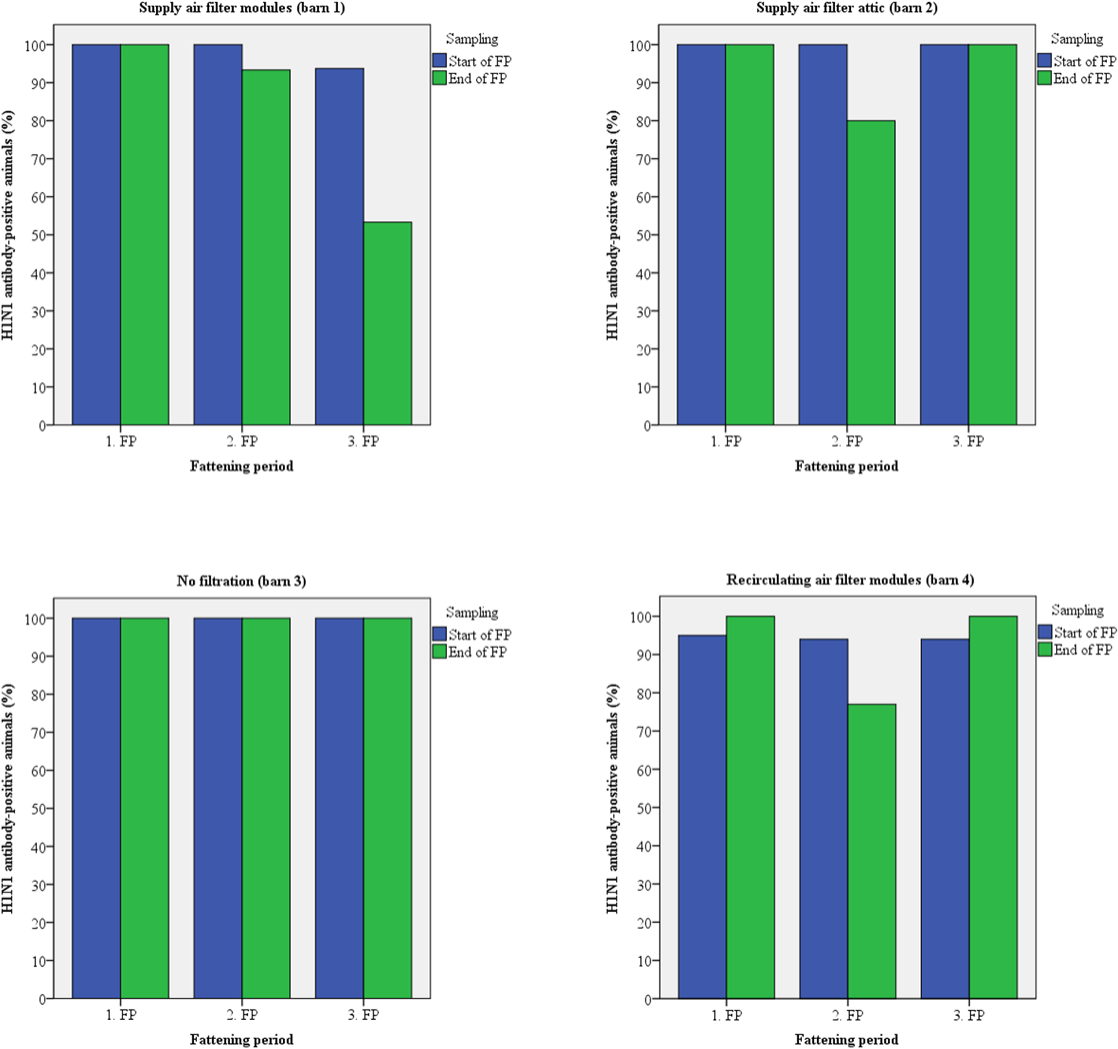
Prevalence of antibodies against H1N1 virus in pigs kept with and without air filtration.

**Fig 6:**
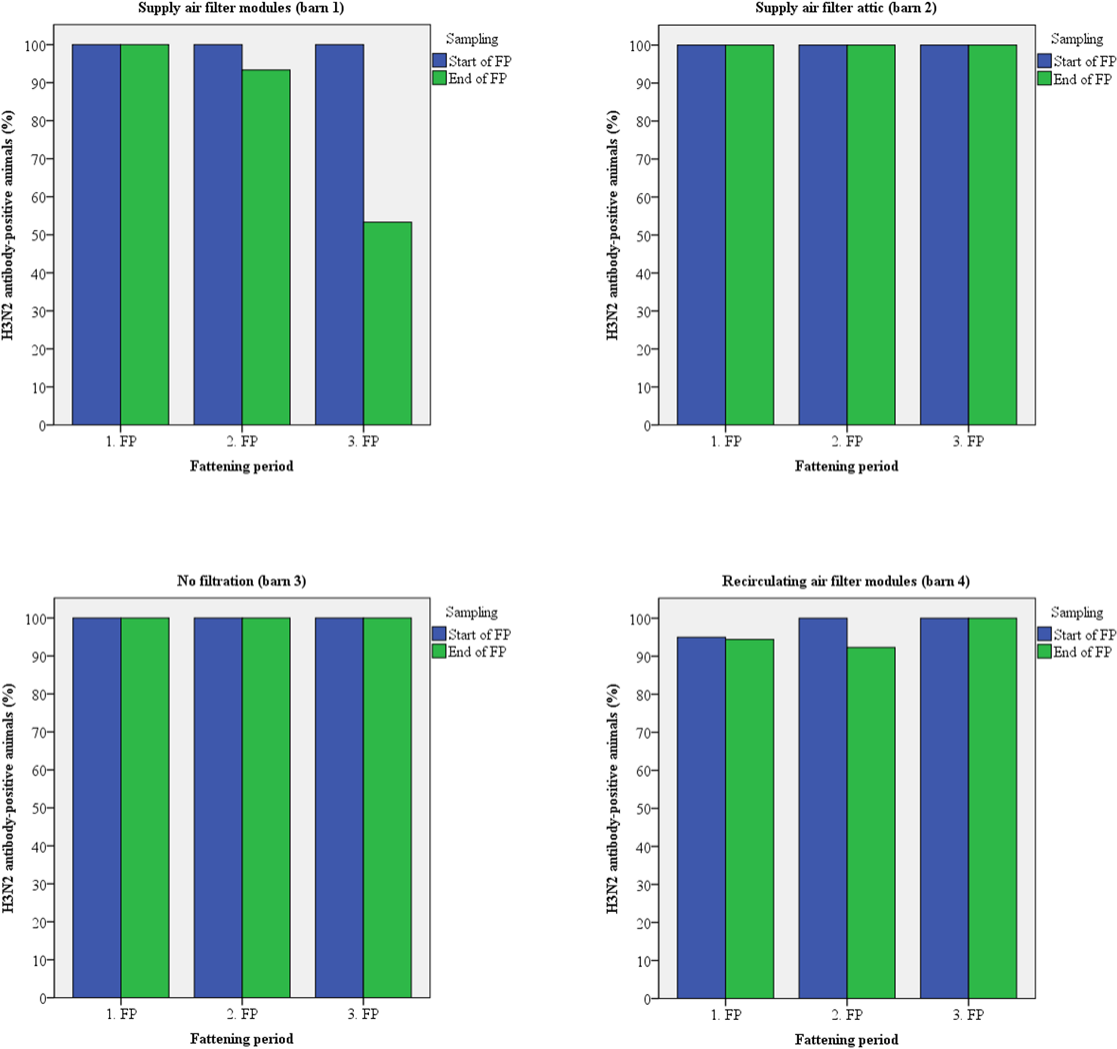
Prevalence of antibodies against H3N2 virus in pigs kept with and without air filtration.

**Fig 7:**
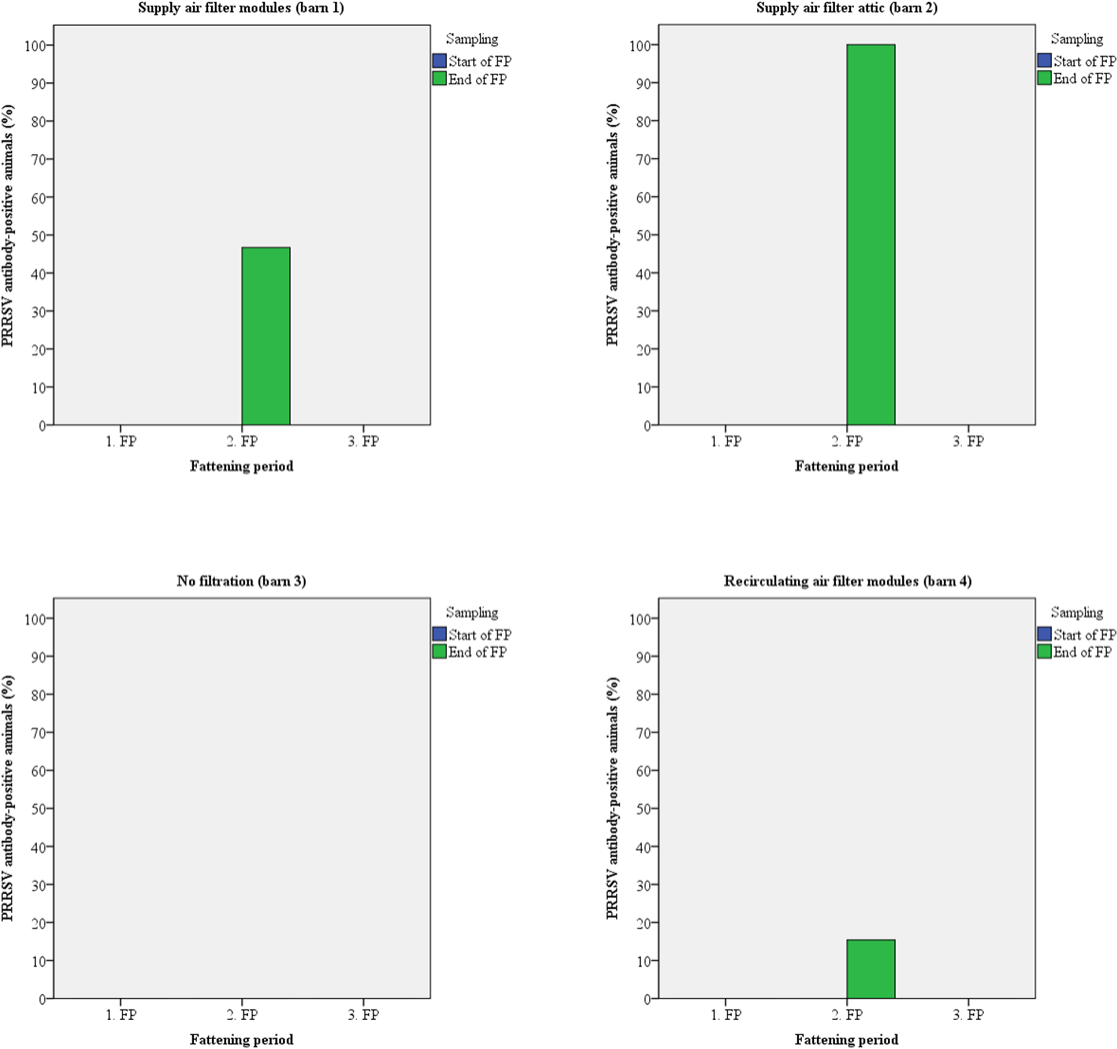
Prevalence of antibodies against PRRSV in pigs kept with and without air filtration.

**Fig 8:**
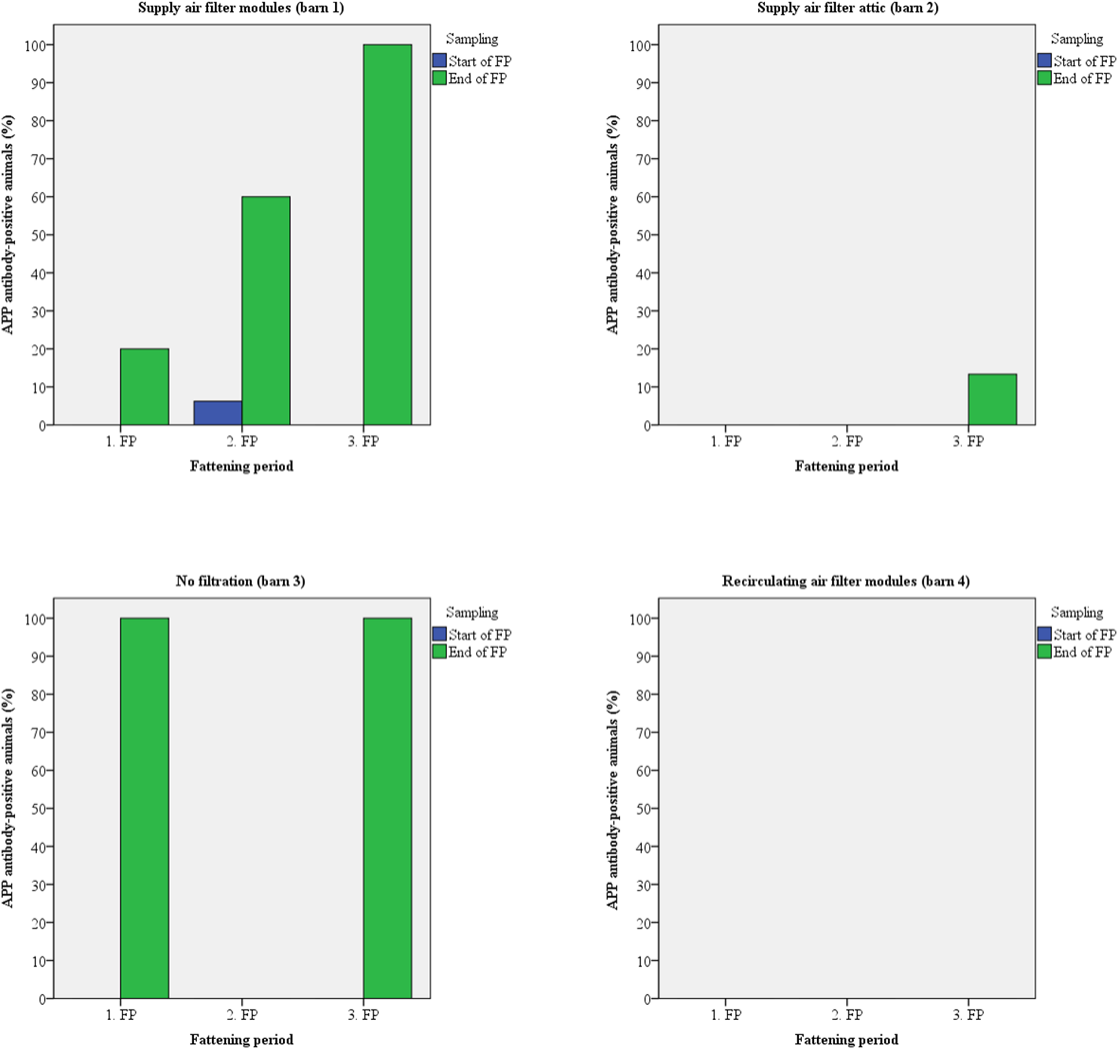
Prevalence of antibodies against APP in animals kept with and without air filtration.

Fifteen animals of each barn were sampled twice for the detection of antibodies. Up to 100% of the test candidates were antibody-positive against Influenza A viruses (H1N1/H2N3) at the arrival at the fattening piggery during all fattening periods (Figs 5 and 6). In most cases, seroprevalence was identical at the time of arrival and at the time of transport to the slaughter house. PRRSV antibody-positive animals were detected only at the end of the second fattening period in every barn except barn 3 (Fig 7). Antibodies against APP were found in animals from all barns except barn 4 (recirculating air filtration) with up to 100% positive samples mainly at the end of the fattening periods (Fig 8).

#### Evaluation of carcasses at the slaughter house

The findings documented at the slaughter house are summarized in Table 10 with emphasis on lung health. Animals of barn 4 (recirculating air filtration) overall revealed best results (i.e. a mean score of 2.0). In each barn animal lung health varied between fattening periods. Noticeably in barn 2 with a bad lung health in the first fattening period compared to a very good and medium score in period 2 and 3, respectively.

**Table 10:** Results of carcass evaluation obtained at slaughter.

Lung parameters	Barn 1: Supply air filter modules						Barn 2: Supply air filter attic						Barn 4: Recirculating air filtration modules						Barn 3: Without filtration					
	1. FP		2. FP		3. FP		1. FP		2. FP		3. FP		1. FP		2. FP		3. FP		1. FP		2. FP		3. FP	
	OCC	S	OCC	S	OCC	S	OCC	S	OCC	S	OCC	S	OCC	S	OCC	S	OCC	S	OCC	S	OCC	S	OCC	S
Pneumonia	1.5%	1	11.7%	2	20.9%	2	17.9%	2	0.2%	0	12.2%	2	0.2%	0	2.1%	1	1.9%	1	8.4%	1	7.5%	1	0.9%	0
Pleurisy	13.2%	2	14.7%	2	21.0%	2	30.7%	3	0.9%	0	9.1%	1	0.7%	0	3.4%	1	5.3%	1	16.4%	2	2.2%	1	23.3%	2
Pericarditis	0.7%	0	1.6%	1	8.0%	1	3.2%	1	1.4%	1	10.7%	1	0.5%	0	1.9%	1	3.7%	1	1.4%	1	2.1%	1	1.3%	1
Total	3		5		5		6		1		4		0		3		3		4		3		3	
**Mean**	**4.3 (medium)**						**4.0 (medium)**						**2.0 (good)**						**3.3 (good)**					

FP—fattening period; OCC—occurrence; S–score; 0–1 = very good, 2–3 = good, 4–5 = medium, 6–7 = bad.

### Pathogen load inside the filter after one year in use

Prefilters (supply air modules of barn 1) harbored 9x10^2^ cfu/ml to 2x10^3^ cfu/ml. The secondary filters revealed 2x10^1^ cfu/ml to 8x10^1^ cfu/ml. In the filter matter samples of barn 2 1x10^2^ cfu/ml to 2x10^2^ cfu/ml were found. The germ spectrum included molds and environmental bacteria including aerobic spore-forming Gram-positive species.

## Discussion

We evaluated the impact of three different mechanical air filtrations systems on air quality, animal health and animal performance in comparison to a non-filtered control.This study was conducted in a commercial pig fattening facility over a period of 13 months in order to take possible seasonal variations into account. All air filtration systems were compatible with the already existing ventilation system and easy to handle for the farmer.

Particulates in the air of livestock buildings include a small proportion carried in by the fresh air supply but most of the larger particles and airborne microorganisms originate from inside the buildings [34]. Airborne microorganisms in animal settings have been shown to be associated with skin, mucous membranes, hair, feathers, and feces and approximately 60% of these microorganisms originate from animal sources [35]. The total amount of airborne bacteria measured in all barns was within the range of other studies [11,35–39]. There were no significant differences (p = 0.824) between barns with and without air filtration. The concentration of airborne MRSA was approximately tenfold higher compared to other investigations [11,18,40], which might be due to the different sampling devices, i.e. wet cyclone technique in our study and AGI 30 impinger used by the others. Moreover, we sampled a tenfold higher total air volume. *Enterobacteriaceae* were intermittently detected which is in concordance to other studies [36,41]. The maximum level of total airborne *E*. *coli* (361 cfu/m^3^) was higher than reported by Yuan et al. [42] but much less than levels found by others [41,43]. Generally, the presence of bedding has been linked to the level of airborne *E*. *coli* [44], however pigs in our study were housed on fully slatted floor and litter was also not applied in the pig farms investigated by von Salviati et al. [41]. Lau et al. [10] reported that total bacterial counts were significantly reduced by two different recirculating air filtration systems, a fabric filter system composed of two prefilters and a high efficiency particulate air (HEPA) filter and another filtration system consisting of a prefilter and an electrostatic precipitator. Removal efficiencies were 10–50% (fabric filter system) and 20–52% (electrostatic precipitator) with the latter system being more effective throughout the year. Similar results were demonstrated by Carpenter and Fryer [15]. However, a reduction of airborne bacteria by recirculating air filtration was not supported by our results.

Airborne dust can cause serious health problems for animals and humans [45]. Its composition is mainly determined by its different sources like animals, manure, litter, and feed [16]. Particles of different kind are classified according to their ability to penetrate the respiratory system. At an aerodynamic diameter of ≤5 μm (respirable particles) particles as well as associated hazards are deposited deep into the lungs [16,46]. Over three consecutive fattening periods total airborne dust ranged on average from 0.12–0.14 mg/m^3^. Most of the single measurements were <0.3 mg/m^3^ and only one value exceeded 0.6 mg/m^3^ whereas other studies reported total dust values of ≥1–8.2 mg/m^3^ [35,38,43,47]. This may be attributed to the short sampling time of 10 min at two-week intervals in our study compared to the others. Some authors affirm that the feeding system greatly affected dust levels in piggeries [46,48] with the lowest values associated with wet feeding as applied in our test facility. The fully slatted floor without litter in our piggery also contributed to lower dust formation. Ventilation rate and air filtration have also been described to enhance air quality in livestock housings. Recirculating ventilation combined with dust filtration using a pocket-filter-type air pollution control device (Shaker Dust Collector) was able to minimize respirable dust concentration by 41% in a swine farrowing room determined by gravimetric measurements [49]. Another study demonstrated a significant average reduction of 40% using air filtration and recirculating air filtration by a coarse prefilter and a fine final filter in weaner rooms [9]. Lau et al. [10] showed that room dust levels efficiently decreased by 18–64% (fabric filter system) and 20–66% (electrostatic precipitator) due to recirculating air filtration. These data are not affirmed by our results obtained in two of the three fattening periods. However, airborne dust concentration was lowest (i.e. 0.063 mg/m^3^) in barn 4 with recirculating air filtration in the third fattening period compared to all other measures carried out over the entire study which indicates a certain influence of this air filtration system. However, a comparable effect on airborne bacteria was not found.

Dust concentration in livestock building was related to deposition rates, ventilation, air recirculation flow rate, size, and number of air filtration inlets and outlets, and humidity [10,15]. Under certain conditions, filtered air was shown to be at least as clean as fresh supply air [15]. Our study with four recirculating air filter modules in barn 4 may not have been sufficient to constantly achieve a significant reduction of dust. Moreover, it has been suggested that dust sedimentation largely contributes to reduction of dust from the air and may even be more efficient than expected. Dust clearance by sedimentation is also enhanced through the voids of mesh and slatted floors [15]. These authors concluded that air filtration by dry filters is a feasible method for reducing dust mass and airborne bacteria in a small room like a flat-deck for early weaners. The main difference between these studies [8–10,15,49] and our study is the size of the barn and the number of animals, the latter mainly contributing to the amount of airborne dust and bacteria. There is no threshold for dust in pig husbandry in Germany but health related dust limits have been suggested for swine health [50]. Our dust values were below the recommended threshold of 3.7 mg/m^3^ for total dust and most values (74/80) were also below 0.23 mg/m^3^ the threshold for respirable particles [50].

Aerial pollutants can distinctly influence animal health, particularly respiratory health. Hence, reduction of these pollutants would improve air quality, animal health and productivity. The effect of air filtration on pig productivity has been evaluated inconsistently and varied from enhanced productivity in large sow herds [7] and earlier marketable state in fattening pigs [8,10] to no positive effects on performance of the pigs [9]. These contradictory results might be a consequence of different management regimes, ventilation systems and various influential factors, which make results difficult to interpret [51]. Regarding pig performance parameters there were only minor differences between the four barns in the study at hand. Noticeably, mortality was lower in barn 1 compared to the other barns. Overall, mortality was predominantly due to non-lung related conditions. Respiratory health was best in animals from barn 4 with recirculating air filtration as evidenced by less sneezing and coughing which was supported by the findings at slaughter (Table 10). This is in concordance to others who found improved pig lung health demonstrated by reduction in lung and snout scores [10]. Barn 4 equipped with four recirculating air filter modules revealed the lowest mean dust concentration. Hence, although these differences were not significant our findings indicate a positive effect of recirculating air filtration on animal lung health. Concentrations of CO_2_ and NH_3_ as well as RH did not differ between barns and could be regarded as ancillary with regard to air quality improvement using air filtration.

The risk of airborne disease transmission will directly be reduced by reducing the number of airborne particles [52]. Regarding within-unit transmission, air filtration combined with positive pressure ventilation has been shown to reduce aerosols [8] and filtration of supply air efficiently reduced PRRSV infection in breeding pig herds [53]. Therefore, pigs were investigated for antibodies against Influenza A viruses, PRRSV, and APP as relevant infectious airborne pathogens [51]. Antibodies against all pathogens were detected but prevalence varied between barns and fattening periods with the exception of Influenza A viruses. The majority of pigs had contact to these viruses prior to stocking (Figs 5 and 6). PRRSV-antibodies were found only at the end of the second fattening period with the highest prevalence in those barns equipped with supply air filtration whereas animals of barn 3 (without any filtration) were negative. Most probably animals had contact to PRRSV just before stocking and the samples that were taken a few days after stocking were still antibody-negative, or the virus was later inadvertently introduced. No PRRSV was detected in any air sample by real-time RT-PCR possibly because there was no virus in the air at the time of sampling or the number of viral particles was below the detection limit of the PCR. Regarding antibodies against APP, barn 4 had no antibody-positive animals over the whole study period. Pigs from barn 1 were positive in all three fattening periods and seropositive animals were also detected in barn 2 (third fattening period) and 3 (first and third fattening period). Antibodies were mainly detected at the end of the respective fattening period as has also been noticed for PRRSV-antibodies and similar explanations might be suggested.

The present study reflects the situation at a commercial fattening piggery which cannot be regarded as equivalent to an experimental test facility. Staff at our test facility must not change clothes between the four barns which may facilitate carryover of diseases and can be seen as a main weakness of this study. Moreover, employees are allowed to enter these barns without showering even if they have been at the piglet rearing unit before which also may have contributed to disease transmission between facilities. Due to work routine this regime could not be changed during our short term project. Furthermore, the unknown health status of piglets at stocking can be regarded as another unpredictable risk factor limiting the outcome of our study. Nevertheless, we demonstrated that supply air filtration as well as recirculating air filtration technique can easily be implemented in an already existing ventilation system and recirculating air filtration positively affects animal lung health. This effect might be enhanced by a combined UVC-light decontamination and recirculating air filtration module. A more prominent effect might have been obtained in a breeding facility because of the longer life span of sows and a higher biosecurity level with air filtration as an add-on measure. In addition we could show that accumulation or multiplication of microorganisms inside the filter materials does not occur suggesting that there is no need to implement inactivation procedures for filters.

## Conclusion

In conclusion, recirculating air filtration resulted in enhanced lung health compared to supply air-filtered and non-filtered barns although we were not able to demonstrate a significant reduction of dust levels and airborne bacteria. In contrast to experimental studies, our study in the field was not able to demonstrate that supply air filtration reduces the risk of introducing airborne transmitted pathogens to animal housings.

## Supporting information

S1 Table Technical information of the filter prototypes. (DOCX)

S2 Table Air quality in barn 1 equipped with supply air filter modules. (PDF)

S3 Table Air quality in barn 2 equipped with a supply air filter attic. (PDF)

S4 Table Air quality in barn 4 equipped with recirculating air filtration modules. (PDF)

S5 Table Air quality in barn 3 without air filtration. (PDF)
