# Supplementary material for: Impact of different supply air and recirculating air filtration systems on stable climate, animal health, and performance of fattening pigs in a commercial pig farm
Source: PLoS One. 2018 Mar 20;13(3):e0194641. doi: 10.1371/journal.pone.0194641 (PMC5860761; doi:10.1371/journal.pone.0194641)
Supplement: S1 Table — (DOCX) [file pone.0194641.s001.docx]

**S1 Table. Technical information of the filter prototypes.**

| **Characteristic** | **Filter in barn 1 (supply air filter modules)** | | **Filter in barn 2 (supply air filter attic)** | **Filter in barn 4 (recirculating air filter modules)** |
| --- | --- | --- | --- | --- |
|  | **Prefilter** | **Secondary filter** |  |  |
| **Description** | Panel filter | Compact filter | Filter wool with glass fiber | Pocket filter |
| **Filter matter** | Polyester | Glass fiber | Glass wool | Polyester |
|  | Thickness 3 mm | Thickness 0.55 mm | Thickness 2 x 40 mm | Thickness 4 mm |
|  | Base Weight 200 g/m² | Base Weight 68 g/m² | Base Weight 1,140 g/m² | Base Weight 120 g/m² |
| **Size (mm)** | 592 x 592 x 48 | 592 x 592 x 292 | 1,200 x 1,200 | 592 x 592 x 360 |
| **Filter surface** | 1.2 m² | 18.8 m² | 1.35 m² | 2.8 m² |
| **Initial pressure loss** | 70 Pa at 2.7 m/s | 110 Pa at 2.7 m/s | 50 Pa at 0.1 m/s | 30 Pa at 0.34 m/s |
| **EU EN 779 class** | G4 | F9 | approx. F8 – F9 | G3 |
| **US ASHRAE standard 52.2-2007** | MERV 6-8^#^ | MERV 16 | MERV 14-16 | MERV 5-6 |

^#^ MERV - minimum efficiency reported value
