# Supplementary material for: Impact of different supply air and recirculating air filtration systems on stable climate, animal health, and performance of fattening pigs in a commercial pig farm
Source: PLoS One. 2018 Mar 20;13(3):e0194641. doi: 10.1371/journal.pone.0194641 (PMC5860761; doi:10.1371/journal.pone.0194641)
Supplement: S2 Table — (PDF) [file pone.0194641.s002.pdf]

S2 Table. Air quality in barn 1 equipped with supply air filter modules.

FP	Season	Week	No. of animals	°C indoors ± SD	°C outdoors ± SD	RH (% ± SD)	Ventilation flow (m^3/h ± SD)	CO_2 (ppm ± SD)	NH_3 (ppm ± SD)	PM 1 (mg/m^3 ± SD)	PM 2.5 (mg/m^3 ± SD)	PM 4 (mg/m^3 ± SD)
1	Autumn	0	0	26.1 ± 1.7	24.5 ± 2.8	na	na	na	na	0.001 ± 0.001	0.001 ± 0.001	0.001 ± 0.001
		2	952	24.9 ± 0.2	14.0 ± 1.4	na	16,688 ± 745	2,270 ± 147*	na	0.020 ± 0.007	0.022 ± 0.008	0.027 ± 0.01
		4	951	25.2 ± 0.2	14.6 ± 1.2	na	17,682 ± 1,102	2,273 ± 137*	na	0.045 ± 0.011	0.048 ± 0.011	0.052 ± 0.01
		6	945	24.8 ± 0.7	13.6 ± 3.2	na	20,911 ± 3,792	2,724 ± 340*	na	0.065 ± 0.014	0.070 ± 0.016	0.079 ± 0.019
		8	941	23.5 ± 0.2	8.3 ± 0.9	na	16,937 ± 1,102	3,297 ± 541*	25.78 ± 4.9	0.095 ± 0.079	0.112 ± 0.107	0.123 ± 0.111
		10	939	23.6 ± 0.2	8.5 ± 2.1	na	16,937 ± 1,102	3,063 ± 183*	18.65 ± 3.2	0.092 ± 0.054	0.107 ± 0.070	0.127 ± 0.076
		12	876	24.0 ± 0.5	12.8 ± 0.3	na	15,073 ± 9,605	1,931 ± 293*	11.60 ± 2.0	0.049 ± 0.011	0.054 ± 0.013	0.066 ± 0.015
2	Winter	0	0	19.3 ± 8.7	7.8 ± 0.9	na	na	na	na	0.041 ± 0.006	0.041 ± 0.006	0.041 ± 0.007
		2	na	na	na	na	na	na	na	na	na	na
		4	945	23.8 ± 0.1	5.3 ± 0.7	66 ± 2	15,620 ± 754	2,270 ± 153	16.95 ± 4.0	0.067 ± 0.025	0.075 ± 0.028	0.092 ± 0.035
		6	944	23.9 ± 0.2	7.4 ± 0.5	69 ± 2	18,303 ± 641	2,197 ± 956	14.25 ± 1.8	0.086 ± 0.016	0.094 ± 0.018	0.111 ± 0.024
		8	940	24.3 ± 0.2	9.0 ± 1.2	64 ± 3	17,309 ± 1,183	2,107 ± 181	19.80 ± 2.1	0.052 ± 0.009	0.058 ± 0.01	0.071 ± 0.011
	Spring	10	938	23.9 ± 0.3	11.8 ± 0.8	66 ± 2	18,303 ± 849	1,583 ± 55	16.60 ± 3.4	0.081 ± 0.049	0.09 ± 0.055	0.11 ± 0.069
		12	802	22.7 ± 0.1	4.9 ± 2.2	72 ± 4	13,831 ± 625	2,744 ± 447	17.75 ± 1.2	0.098 ± 0.007	0.107 ± 0.006	0.125 ± 0.004
3	Spring	0	0	23.3 ± 0.5	13.1 ± 0.9	na	na	na	17.4 ± 5.0	0.008 ± 0	0.008 ± 0	0.008 ± 0
		2	956	25.1 ± 0.6	10.8 ± 2.0	66 ± 5	12,838 ± 1,463	2,540 ± 343	9.10 ± 1.8	0.075 ± 0.025	0.083 ± 0.028	0.101 ± 0.035
		4	952	25.4 ± 0.6	15.2 ± 2.4	62 ± 6	19,794 ± 1,924	1,850 ± 256	na	0.049 ± 0.009	0.053 ± 0.01	0.063 ± 0.013
		6	951	23.7 ± 0.3	10.0 ± 0.9	69 ± 3	15,322 ± 811	2,140 ± 166	na	0.067 ± 0.003	0.074 ± 0.004	0.090 ± 0.004
	Summer	8	943	28.9 ± 0.4	25.6 ± 0.6	68 ± 1	32,712 ± 248	1,409 ± 99	na	0.034 ± 0.006	0.036 ± 0.007	0.040 ± 0.008
		10	938	25.7 ± 0.3	18.9 ± 2.1	73 ± 2	26,750 ± 1,307	1,446 ± 156	12.45 ± 0.6	0.042 ± 0.012	0.044 ± 0.013	0.049 ± 0.015
		12	895	26.5 ± 0.2	22.8 ± 1.6	71 ± 2	36,190 ± 1,307	1,214 ± 65	26.80 ± 2.1	0.035 ± 0.025	0.038 ± 0.027	0.047 ± 0.035

Measurements were taken at two week intervals and sampling was performed between 9 a.m. and 12 p.m.. Temperatures, relative humidity (RH), ventilation flow, and CO_2 represent the mean of data recorded during sampling time.
Dust values were calculated from data collected by the DustTrak™ DRX Aerosol Monitor over 10 min at two sampling points. NH3 values are means of two measurements per barn.
Seasons were defined according to the astronomical calendar: spring (21st March to 20th June), summer (21st June to 22nd September), autumn (23rd September to 21st December), winter (22nd December to 20th March).
*measured with a handheld device (Testo 535); FP - fattening period; na - data not available; SD - standard deviation; PM - particulate matter
