# Supplementary material for: Impact of different supply air and recirculating air filtration systems on stable climate, animal health, and performance of fattening pigs in a commercial pig farm
Source: PLoS One. 2018 Mar 20;13(3):e0194641. doi: 10.1371/journal.pone.0194641 (PMC5860761; doi:10.1371/journal.pone.0194641)
Supplement: S3 Table — (PDF) [file pone.0194641.s003.pdf]

S3 Table. Air quality in barn 2 equipped with a supply air filter attic.

FP	Season	Week	No. of animals	°C indoors ± SD	°C outdoors ± SD	RH (% ± SD)	Ventilation flow (m^3/h ± SD)	CO_2 (ppm ± SD)	NH_3 (ppm ± SD)	PM 1 (mg/m^3 ± SD)	PM 2.5 (mg/m^3 ± SD)	PM 4 (mg/m^3 ± SD)
1	Autumn	0	0	na	na	na	na	na	na	0.001 ± 0.002	0.001 ± 0.002	0.001 ± 0.003
		2	954	28.4 ± 0.9	25.2 ± 2.5	na	34,978 ± 4,330	1,486 ± 202*	na	0.014 ± 0.003	0.015 ± 0.003	0.015 ± 0.003
		4	952	27.6 ± 1.5	24.5 ± 2.8	na	38,383 ± 1,589	1,707 ± 718*	na	0.016 ± 0.006	0.017 ± 0.007	0.021 ± 0.008
		6	945	24.0 ± 0.3	14.0 ± 1.4	na	18,176 ± 1,251	2,379 ± 100*	na	0.046 ± 0.016	0.050 ± 0.017	0.060 ± 0.02
		8	934	24.9 ± 0.2	14.6 ± 1.2	na	25,214 ± 936	2,484 ± 145*	na	0.046 ± 0.018	0.050 ± 0.02	0.057 ± 0.026
		10	928	23.8 ± 0.6	13.6 ± 3.2	na	24,397 ± 4,395	2,549 ± 118*	na	0.077 ± 0.021	0.081 ± 0.024	0.090 ± 0.03
		12	912	23.1 ± 0.5	8.3 ± 0.9	na	21,581 ± 3,176	2,902 ± 302*	31.62 ± 4.9	0.060 ± 0.023	0.064 ± 0.026	0.073 ± 0.032
2	Winter	0	0	na	na	na	na	na	na	na	na	na
		2	960	24.4 ± 0.3	7.3 ± 0.4	na	15,542 ± 1,854	3,242 ± 296*	15.90 ± 1.7	0.057 ± 0.001	0.063 ± 0	0.078 ± 0
		4	954	24.3 ± 0.1	1.4 ± 1.5	na	15,315 ± 1,529	4,363 ± 315*	15.90 ± 2.5	0.343 ± 0.37	0.435 ± 0.488	0.543 ± 0.622
		6	na	na	na	na	na	na	na	na	na	na
		8	945	23.3 ± 0.4	4.2 ± 0.4	74 ± 3	16,450 ± 1,304	3,523 ± 138*	26.55 ± 2.1	0.039 ± 0.002	0.043 ± 0.001	0.053 ± 0.003
	Spring	10	942	23.9 ± 0.2	7.4 ± 0.5	80 ± 2	19,856 ± 945	3,208 ± 204*	23.80 ± 7.1	0.089 ± 0.016	0.097 ± 0.018	0.116 ± 0.023
		12	891	23.4 ± 0.4	9.0 ± 1.2	72 ± 3	18,039 ± 1,979	3,010 ± 184*	29.95 ± 7.1	0.031 ± 0.016	0.036 ± 0.018	0.044 ± 0.023
3	Spring	0	0	16.4 ± 1.4	4.9 ± 2.2	70 ± 1	na	2,150 ± 454	na	0.143 ± 0.003	0.144 ± 0.002	0.144 ± 0.002
		2	944	25.1 ± 0.1	8.0 ± 1.0	76 ± 3	15,996 ± 642	3,083 ± 336	11.50 ± 3.4	0.137 ± 0.129	0.175 ± 0.178	0.203 ± 0.206
		4	942	24.4 ± 0.2	13.1 ± 0.9	71 ± 3	18,721 ± 435	1,749 ± 233	16.35 ± 1.3	0.038 ± 0.001	0.040 ± 0.001	0.045 ± 0.002
		6	939	24.3 ± 0.4	10.8 ± 2.0	71 ± 3	20,083 ± 1,940	2,364 ± 101	24.5 ± 7.2	0.034 ± 0.005	0.038 ± 0.005	0.046 ± 0.005
	Summer	8	936	24.7 ± 1.0	15.2 ± 2.4	66 ± 6	26,440 ± 3,327	1,990 ± 316	na	0.030 ± 0.006	0.032 ± 0.007	0.037 ± 0.008
		10	933	22.6 ± 0.2	10.0 ± 1.0	72 ± 1	19,629 ± 642	2,114 ± 110	na	0.052 ± 0.027	0.058 ± 0.03	0.071 ± 0.037
		12	847	29.0 ± 0.9	25.6 ± 0.6	71 ± 1	36,431 ± 0	1,130 ± 209	na	0.038 ± 0.009	0.040 ± 0.011	0.045 ± 0.014

Measurements were taken at two week intervals and sampling was performed between 9 a.m. and 12 p.m.. Temperatures, relative humidity (RH), ventilation flow, and CO_2 represent the mean of data recorded during sampling time.
Dust values were calculated from data collected by the DustTrak™ DRX Aerosol Monitor over 10 min at two sampling points. NH3 values are means of two measurements per barn.
Seasons were defined according to the astronomical calendar: spring (21st March to 20th June), summer (21st June to 22nd September), autumn (23rd September to 21st December), winter (22nd December to 20th March).
*measured with a handheld device (Testo 535); FP - fattening period; na - data not available; SD - standard deviation; PM - particulate matter
