# Supplementary material for: Impact of different supply air and recirculating air filtration systems on stable climate, animal health, and performance of fattening pigs in a commercial pig farm
Source: PLoS One. 2018 Mar 20;13(3):e0194641. doi: 10.1371/journal.pone.0194641 (PMC5860761; doi:10.1371/journal.pone.0194641)
Supplement: S4 Table — (PDF) [file pone.0194641.s004.pdf]

S4 Table. Air quality in barn 4 equipped with recirculating air filtration modules.

FP	Season	Week	No. of animals	°C indoors ± SD	°C outdoors ± SD	RH (% ± SD)	Ventilation flow (m^3/h ± SD)	CO_2 (ppm ± SD)	NH_3 (ppm ± SD)	PM 1 (mg/m^3 ± SD)	PM 2.5 (mg/m^3 ± SD)	PM 4 (mg/m^3 ± SD)
1	Autumn	0	0	20.1[§]	9.2[§]	na	na	na	na	0.096 ± 0.007	0.096 ± 0.008	0.097 ± 0.008
		2	960	24.3[§]	10.0[§]	na	12,976[§]	3,726 ± 190*	12.33 ± 0.8	0.110 ± 0.009	0.113 ± 0.01	0.120 ± 0.012
		4	960	24.5 ± 0.2	12.8 ± 0.3	na	17,707 ± 1,745	2,050 ± 163*	10.55 ± 0.1	0.036 ± 0.009	0.039 ± 0.01	0.045 ± 0.013
		6	944	23.9 ± 0.3	8.8 ± 0.6	na	17,594 ± 999	2,698 ± 73*	17.05 ± 0.8	0.059 ± 0.009	0.065 ± 0.01	0.077 ± 0.013
		8	943	23.5 ± 0.3	7.8 ± 0.9	na	17,144 ± 1,489	3,142 ± 549*	15.90 ± 0.4	0.163 ± 0.045	0.173 ± 0.049	0.194 ± 0.055
		10	na	na	na	na	na	na	na	na	na	na
		12	832	22.8 ± 0.4	4.2 ± 0.8	71 ± 2	16,581 ± 1,185	2,638 ± 245*	26.75 ± 7.4	0.076 ± 0.022	0.084 ± 0.024	0.102 ± 0.03
2	Winter	0	0	19.7 ± 0.1	5.7 ± 1.0	na	na	na	16.55 ± 7.4	0.039 ± 0.011	0.039 ± 0.011	0.040 ± 0.012
		2	939	24.6 ± 0.2	4.3 ± 0.7	71 ± 2	14,553 ± 863	2,752 ± 171	8.65 ±5.2	0.041 ± 0.013	0.047 ± 0.015	0.056 ± 0.018
		4	934	23.7[§]	7.9[§]	66[§]	20,185[§]	2,111[§]	14.95 ± 3.9	0.097 ± 0.022	0.103 ± 0.024	0.118 ± 0.03
		6	931	23.9 ± 0.2	7.2 ± 0.8	69 ± 3	16,806 ± 260	2,351 ± 84	12.45 ± 2.3	0.055 ± 0.006	0.060 ± 0.006	0.068 ± 0.007
		8	928	25.3 ± 0.3	14.8 ± 2.1	66 ± 4	24,917 ± 1,073	1,676 ± 317	19.65 ± 2.2	0.102 ± 0.025	0.109 ± 0.028	0.124 ± 0.034
	Spring	10	926	23.5 ± 0.3	8.5 ± 1.0	65 ± 4	17,707 ± 1,192	1,830 ± 177	24.35 ± 0.1	0.072 ± 0.021	0.079 ± 0.024	0.095 ± 0.029
		12	832	23.4 ± 0.8	9.8 ± 1.0	68 ± 2	20,321 ± 3,848	1,818 ± 193	n.a.	0.052 ± 0.002	0.057 ± 0.002	0.066 ± 0.003
3	Spring	0	0	25.0 ± 0.1	18.5 ± 0.2	na	na	na	na	0.075 ± 0.002	0.075 ± 0.003	0.076 ± 0.002
		2	956	25.3 ± 0.2	17.4 ± 1.1	73 ± 1	18,969 ± 1,185	1,497 ± 35	9.45 ± 1.1	0.026 ± 0.001	0.028 ± 0.002	0.032 ± 0.002
		4	950	25.8 ± 0.5	19.8 ± 1.6	68 ± 3	24,917 ± 2,341	1,497 ± 64	14.70 ± 1.6	0.036 ± 0.016	0.039 ± 0.017	0.045 ± 0.02
		6	948	31.6 ± 1.0	29.4 ± 1.5	64 ± 3	40,012 ± 0	1,199 ± 46	13.50 ± 1.1	0.031 ± 0.008	0.032 ± 0.008	0.034 ± 0.01
	Summer	8	942	26.3 ± 0.5	21.5 ± 0.9	75 ± 1	36,633 ± 1,352	1,250 ± 57	15.55 ± 1.9	0.050 ± 0.009	0.051 ± 0.008	0.054 ± 0.006
		10	931	24.8 ± 0.3	14.8 ± 1.3	74 ± 2	26,854 ± 2,506	1,997 ± 304	25.65 ± 3.9	0.028 ± 0.008	0.031 ± 0.009	0.035 ± 0.011
		12	916	27.9 ± 1,3	24.0 ± 1.8	70 ± 3	35,416 ± 225	1,334 ± 58	20.35 ± 0.8	0.032 ± 0.008	0.034 ± 0.009	0.038 ± 0.01

Measurements were taken at two week intervals and sampling was performed between 9 a.m. and 12 p.m.. Temperatures, relative humidity (RH), ventilation flow, and CO_2 represent the mean of data recorded during sampling time.
Dust values were calculated from data collected by the DustTrak™ DRX Aerosol Monitor over 10 min at two sampling points. NH3 values are means of two measurements per barn.
Seasons were defined according to the astronomical calendar: spring (21st March to 20th June), summer (21st June to 22nd September), autumn (23rd September to 21st December), winter (22nd December to 20th March).

[§]data logging over time by the computer system failed and the given value represents a single value taken directly from the control panel in front of the barn

*measured with a handheld device (Testo 535); FP - fattening period; na - data not available; SD - standard deviation; PM - particulate matter

[#]due to organizational reasons sampling at the abandoned barn was not possible
