# Supplementary material for: Impact of different supply air and recirculating air filtration systems on stable climate, animal health, and performance of fattening pigs in a commercial pig farm
Source: PLoS One. 2018 Mar 20;13(3):e0194641. doi: 10.1371/journal.pone.0194641 (PMC5860761; doi:10.1371/journal.pone.0194641)
Supplement: S5 Table — (PDF) [file pone.0194641.s005.pdf]

S5 Table. Air quality in barn 3 without air filtration.

FP	Season	Week	No. of animals	°C indoors ± SD	°C outdoors ± SD	RH (% ± SD)	Ventilation flow (m^3/h ± SD)	CO_2 (ppm ± SD)	NH_3 (ppm ± SD)	PM 1 (mg/m^3 ± SD)	PM 2.5 (mg/m^3 ± SD)	PM 4 (mg/m^3 ± SD)
1	Autumn	0	0	23.7 ± 1.2	14.3 ± 0.7	na	na	na	na	0.015 ± 0.01	0.016 ± 0.01	0.016 ± 0.01
		2	943	25.8 ± 0.2	15.4 ± 0.2	na	22,145 ± 1,398	2,115 ± 75*	na	0.106 ± 0.034	0.107 ± 0.035	0.110 ± 0.036
		4	940	24.4 ± 0.1	9.2 ± 0.6	na	16,644 ± 889	2,758± 281*	14.78 ± 0.9	0.103 ± 0.008	0.105 ± 0.008	0.110 ± 0.008
		6	938	25.2[§]	9.5[§]	na	24,433[§]	3,590 ± 408*	19.95 ± 2.8	0.185 ± 0.109	0.193 ± 0.118	0.203 ± 0.121
		8	931	24.3 ± 0.3	12.8 ± 0.3	na	25,309 ± 1,838	2,051 ± 185*	13.65 ± 0.9	0.06 ± 0.014	0.064 ± 0.015	0.073 ± 0.016
		10	916	24.1 ± 0.5	8.8 ± 0.6	na	23,119 ± 2,677	2,793 ± 272*	21.05 ± 1.5	0.079 ± 0.02	0.088 ± 0.023	0.105 ± .003
		12	861	23.1 ± 0.3	1.4 ±1.5	na	16,888 ± 1,159	4,024 ± 222*	27.90 ± 7.2	0.106 ± 0.033	0.117 ± 0.036	0.138 ± 0.044
2	Winter	0	0	19.0 ± 2.4	4.5 ± 0.3	na	na	na	na	0.023 ± 0.004	0.024 ± 0.005	0.024 ± 0.005
		2	941	25.1 ± 0.2	11.2 ± 1.1	67 ± 3	17,374 ± 487	1,995 ± 166	12.55 ± 0.6	0.056 ± 0.005	0.060 ± 0.006	0.071 ± 0.008
		4	936	24.2 ± 0.2	5.7 ± 1.0	67 ± 3	15,573 ± 243	2,679 ± 143	14.05 ± 1.8	0.060 ± 0.035	0.066 ± 0.038	0.078 ± 0.047
		6	928	24.1 ± 0.2	4.3 ± 0.7	75 ± 3	15,330 ± 466	3,187 ± 196	18.1 ± 1.4	0.058 ± 0.009	0.065 ± 0.011	0.08 ± 0.015
		8	926	23.8[§]	5.3[§]	76[§]	15,671[§]	2,974[§]	24.25 ± 5.3	0.075 ± 0	0.078 ± 0.001	0.084 ± 0.001
	Spring	10	924	23.2 ± 0.1	7.2 ± 0.8	75 ± 1	17,277 ± 613	2,690 ± 158	19.40 ± 8.3	0.065 ± 0.001	0.071 ± 0.001	0.082 ± 0.002
		12	894	25.0 ± 0.2	14.8 ± 2.1	69 ± 2	27,597 ± 1,225	1,957 ± 176	22.80 ± 2.1	0.098 ± 0.013	0.104 ± 0.015	0.117 ± 0.018
3	Spring	0	600[#]	24.4 ± 0.1	9.8 ± 1.1	na	na	na	na	0.065 ± 0.023	0.076 ± 0.028	0.089 ± 0.035
		2	953	25.9 ± 0.1	16.2 ± 2.3	73 ± 3	15,305 ± 466	2,411 ± 382	na	0.087 ± 0.042	0.096 ± 0.05	0.101 ± 0.049
		4	950	26.7 ± 0.1	18.5 ± 0.2	76 ± 1	25,285 ± 1,004	1,644 ± 115	na	0.083 ± 0.007	0.084 ± 0.007	0.086 ± 0.007
		6	948	25.8 ± 0.3	17.4 ± 1.1	73 ±1	26,624 ± 1,288	1,778 ± 140	15,9 ± 5.4	0.031 ± 0.015	0.033 ± 0.017	0.039 ± 0.021
	Summer	8	945	25.8 ± 0.5	19.8 ± 1.6	67 ± 4	31,492 ± 3,192	1,454 ± 161	25.60 ± 5.1	0.034 ± 0	0.037 ± 0	0.043 ± 0
		10	938	31.7 ± 0.8	29.4 ± 1.5	63 ± 3	40,985 ± 0	1,164 ± 64	15.90 ± 2.8	0.038 ± 0.003	0.039 ± 0.003	0.042 ± 0.004
		12	932	27.2 ± 0.5	21.5 ± 0.9	75 ± 2	39,037 ± 0	1,287 ± 190	21,80 ± 0	0.073 ± 0.001	0.074 ± 0	0.077 ± 0.001

Measurements were taken at two week intervals and sampling was performed between 9 a.m. and 12 p.m.. Temperatures, relative humidity (RH), ventilation flow, and CO_2 represent the mean of data recorded during sampling time.
Dust values were calculated from data collected by the DustTrak™ DRX Aerosol Monitor over 10 min at two sampling points. NH3 values are means of two measurements per barn.
Seasons were defined according to the astronomical calendar: spring (21st March to 20th June), summer (21st June to 22nd September), autumn (23rd September to 21st December), winter (22nd December to 20th March).
[§]data logging over time by the computer system failed and the given value represents a single value taken directly from the control panel in front of the barn
*measured with a handheld device (Testo 535); FP - fattening period; na - data not available; SD - standard deviation; PM - particulate matter
[#]due to organizational reasons sampling at the abandoned barn was not possible
